# Caffeine regulates both osteoclast and osteoblast differentiation via the AKT, NF-κB, and MAPK pathways

**DOI:** 10.3389/fphar.2024.1405173

**Published:** 2024-06-13

**Authors:** Yue Miao, Lei Zhao, Shuwen Lei, Chunyan Zhao, Qiuping Wang, Chao Tan, Chunxiu Peng, Jiashun Gong

**Affiliations:** ^1^ College of Food Science and Technology, Yunnan Agricultural University, Kunming, Yunnan, China; ^2^ Medicinal Plants Research Institute, Yunnan Academy of Agricultural Sciences, Kunming, Yunnan, China; ^3^ College of Science, Yunnan Agricultural University, Kunming, China; ^4^ College of Horticulture and Landscape, Yunnan Agricultural University, Kunming, China; ^5^ Agro-Products Processing Research Institute, Yunnan Academy of Agricultural Sciences, Kunming, China

**Keywords:** caffeine, osteoclasts, osteoblasts, bone metabolism, ovariectomy

## Abstract

**Background:** Although caffeine generally offers benefits to human health, its impact on bone metabolism remains unclear.

**Aim and Methods:** This study aimed to systematically evaluate the long-term effects of caffeine administration on osteoclasts, osteoblasts, and ovariectomy-induced postmenopausal osteoporosis (OP).

**Results:** Our *in vitro* findings revealed that 3.125 and 12.5 μg/mL caffeine inhibited RANKL-mediated osteoclastogenesis in RAW 264.7 cells through the MAPK and NF-κB pathways, accompanied by the inactivation of nuclear translocation of nuclear factor NFATc1. Similarly, 3.125 and 12.5 μg/mL of caffeine modulated MC3T3-E1 osteogenesis via the AKT, MAPK, and NF-κB pathways. However, 50 μg/mL of caffeine promoted the phosphorylation of IκBα, P65, JNK, P38, and AKT, followed by the activation of NFATc1 and the inactivation of Runx2 and Osterix, ultimately disrupting the balance between osteoblastogenesis and osteoclastogenesis. *In vivo* studies showed that gavage with 55.44 mg/kg caffeine inhibited osteoclastogenesis, promoted osteogenesis, and ameliorated bone loss in ovariectomized mice.

**Conclusion:** Conversely, long-term intake of high-dose caffeine (110.88 mg/kg) disrupted osteogenesis activity and promoted osteoclastogenesis, thereby disturbing bone homeostasis. Collectively, these findings suggest that a moderate caffeine intake (approximately 400 mg in humans) can regulate bone homeostasis by influencing both osteoclasts and osteoblasts. However, long-term high-dose caffeine consumption (approximately 800 mg in humans) could have detrimental effects on the skeletal system.

## 1 Introduction

Osteoporosis (OP) is a systemic metabolic disease characterized by low bone mass and microarchitectural deterioration of bone tissue. This combination leads to increased bone fragility and a propensity for fractures. In the context of the world’s progressively aging population, osteoporosis has become a major public health concern (Erzsébet et al., 2018). Several factors reportedly contribute to osteoporosis, including hormone fluctuations, nutrition, inflammatory stress, and mechanical stress (Wang et al., 2017). Currently, various pharmacological agents are used to treat osteoporosis; however, these drugs may have adverse effects on human health (Tabatabaei-Malazy et al., 2017). Therefore, lifestyle modifications are recommended to address unhealthy behaviors, such as excessive caffeine intake, that may contribute to osteoporosis.

Caffeine (1,3,7-trimethylxanthine) is the most widely used and studied pharmacologically active substance in the world. It is found in foods, beverages, and medicinal preparations, such as coffee, tea, soft drinks, cocoa or chocolate, and various medications and dietary supplements (Liu et al., 2017). Current evidence suggests that approximately 90% of adults regularly consume caffeine-containing foods and beverages throughout their lives (Reuter et al., 2021a). Numerous studies have shown that caffeine can stimulate the brain and nervous system (Tad et al., 2010), improve memory and cognition (Dam et al., 2020), and even decrease the risk of type-2 diabetes by promoting insulin sensitivity (Said et al., 2020).

Meanwhile, some epidemiological studies have suggested that caffeine intake accelerates calcium loss, potentially increasing the risk of bone loss and osteoporosis (Hallström et al., 2006; Nash and Ward, 2015). Researchers have also found that caffeine can stimulate bone resorption in two ways: by promoting osteoclast differentiation and maturation (Choi et al., 2013) and by affecting the osteogenic differentiation and bone-forming ability of bone marrow-derived mesenchymal stromal cells (BMMSCs) (Su et al., 2013). When the activities of osteoclasts and osteoblasts are dysregulated, bone metabolism is negatively affected (Reis et al., 2016). Animal studies have yielded inconsistent results, with some studies reporting that high-dose caffeine consumption (diets with 0.2% caffeine or 100 mg/kg) inhibits bone growth, decreases bone mineral density (Liu et al., 2011), and interrupts bone healing (Duarte et al., 2009). Conversely, low to moderate doses (20 mg/kg) have been shown to increase bone mass and strength in ovariectomized rats (Folwarczna et al., 2013). Another study found that long-term administration of a moderate dose (30–120 mg/kg) did not affect the skeletal system in ovariectomized rats (Shangguan et al., 2017). These conflicting findings highlight the need for further research. The data from these studies suggest that exceeding the recommended daily intake of 400 mg of caffeine can lead to net bone loss. In contrast, moderate caffeine intake (less than 400 mg) might play a role in regulating bone metabolism.

Several studies have linked caffeine consumption to osteoporosis, suggesting a potential effect on bone density. Given the widespread use of caffeine, its impact on bone health could be significant. With the increasing pace of work, many people may consume six or more cups of coffee daily. Therefore, investigating whether caffeine can lead to bone loss is crucial. This analysis aimed to examine the impact of caffeine consumption on osteoblasts and osteoclasts, the cells responsible for bone formation and breakdown, respectively. Additionally, the study sought to elucidate the effects of caffeine on overall bone homeostasis and the balance between bone formation and resorption. By understanding the results of this investigation, we may gain a scientific basis for informed consumption of caffeinated beverages.

## 2 Materials and methods

### 2.1 Reagents and animals

#### 2.1.1 Reagents

Caffeine (98% purity, molecular biology, and cell study grade) was obtained from Wei Ke Qi Sheng Wu (Sichuan Province, China). Minimum essential medium alpha (MEM-α), Dulbecco’s modified eagle medium (DMEM), fetal bovine serum (FBS), penicillin–streptomycin, and 0.25% trypsin–EDTA were purchased from Biological Industries (Israel). Phosphate-buffered saline (PBS), RANKL, L-ascorbic acid, β-glycerophosphate, vitamin D3, strontium ranelate, and alizarin S red were obtained from Sigma-Aldrich (St. Louis, MO, United States). Alkaline phosphatase (ALP) assay kits were purchased from Elabscience (Elabscience Biotechnology Co., Ltd., China). Cell Counting Kit-8 (CCK-8) and tartrate-resistant acid phosphatase (TRAP) staining kits were obtained from Proteintech (Wuhan Sanying, China).

#### 2.1.2 Antibodies

Rabbit polyclonal primary antibodies against β-Actin, COL1A1, RUNX2, Osterix, MMP9, NFATc1, c-FOS, TRAP, RANKL CTR, CTSK, IκBɑ, and pIκBɑ were purchased from ABclonal (Boston, United States). Rabbit monoclonal primary antibodies against phosphorylated-AKT (Ser473), AKT, phosphorylated-p44/42 MAPK (ERK1/2), and p44/42 MAPK (ERK1/2), antibodies against phosphorylated-p38 MAPK (Thr180/Tyr182), P38, antibodies against phosphorylated-SAPK/JNK (Thr183/Tyr185), JNK, and antibodies against phosphorylated-NF-κB p65 (Ser536), and NF-κB p65 were purchased from Cell Signaling Technology (Danvers, MA, United States). Goat anti-rabbit IgG (H+L) secondary antibody Alexa Fluor 546 was purchased from molecular probes (Waltham, MA, United States).

#### 2.1.3 Animals

C57BL/6 female mice (8-week-old, weight 20–25 g) were ordered from Skbex Biotechnology (Henan, China) [license no: SCXK (Dian) 2020-0005].

### 2.2 Cell culture

RANKL stimulation of murine monocytic cell lines, RAW 264.7, which are widely believed to be macrophages/pre-osteoclasts, induces their differentiation into bone-absorbing osteoclasts (Wu et al., 2017; Hongyuan et al., 2019). For the present study, RAW 264.7 cells (SCSP-5036) were purchased from the cell library of the Chinese Academy of Sciences and cultured in a growth medium containing DMEM, 10% fetal bovine serum, and penicillin (100 U/mL)/streptomycin (100 μg/mL). The cells were cultured in a 37°C incubator with 5% CO_2_ (Thermo Fisher, Waltham, MA, United States).

RAW 264.7 cells were divided into six groups: a control group treated with 50 ng/mL RANKL only, two positive control groups treated with RANKL plus 50 μg/mL each of VD (Vitamin D) and LN (Strontium Ranelate), and three experimental groups treated with RANKL plus caffeine at doses of 3.125, 12.5, and 50 μg/mL.

A useful model for studying osteoblastic functions is the MC3T3-E1 cell culture system (Choi et al., 2016). Modulating both osteoclast and osteoblast differentiation can regulate bone homeostasis. The mouse pre-osteoblast cell line MC3T3-E1 (subclone 4, ATCC CRL-2593) was purchased from the American Type Culture Collection (ATCC) (Manassas, VA, United States). Once the cells reached approximately 80%–90% confluence, they were then sub-cultured by treatment with 0.25% trypsin–EDTA and grown in appropriate sterile tissue culture plates.

MC3T3-E1 cells were divided into six groups: a control group receiving 50 μg/mL L-ascorbic acid and 10 mM β-glycerophosphate; two positive control groups receiving L-ascorbic acid, β-glycerophosphate, 50 μg/mL VD (Vitamin D), and 50 μg/mL LN (Strontium ranelate); and three experimental groups receiving L-ascorbic acid, β-glycerophosphate, and caffeine at doses of 3.125, 12.5, and 50 μg/mL.

### 2.3 TRAP and ALP assays

RAW 264.7 cells were incubated with the samples for 5 days. After incubation, the cells were fixed with 4% paraformaldehyde and sectioned. Osteoclast activity was then detected using a TRAP staining kit. The stained osteoclasts were quantified using ImageJ software. Additionally, TRAP activity in the cell culture medium on the 5th day was measured using a TRAP assay kit according to the manufacturer’s instructions.

MC3T3-E1 cells were incubated with the samples for 14 days. On the 14th day, ALP activity in the cell culture medium was measured using an alkaline phosphatase assay kit according to the manufacturer’s instructions.

### 2.4 Western blot

After treatment, proteins from the cells were harvested using RIPA lysis buffer and quantified using a Bicinchoninic Acid Protein Assay Kit (Beyotime Biotechnology Inc., Jiangsu, China). The proteins were then separated on 10% polyacrylamide gels and transferred to polyvinylidene fluoride (PVDF) membranes (Millipore, Billerica, MA, United States). These membranes were blocked with 5% BSA in TBST and incubated with the corresponding primary antibodies. Horseradish peroxidase-conjugated anti-rabbit secondary antibodies were diluted at 1:3000 and incubated at room temperature for 1 h. The signals were detected using an ECL Chemiluminescence Kit (7Sea Biotech, Shanghai, China) with a Tanon 5200 Imaging System (Tianneng, Shanghai, China).

### 2.5 Animal experimental designs

All animal experiments were approved by the Institutional Ethics Committee of Yunnan Agricultural University and performed in accordance with the guidelines for the care and use of laboratory animals. The US Food and Drug Administration (FDA) recommends a daily caffeine intake of 400 mg for a person weighing 60 kg (FDA, 2018). Intragastric infusion doses were calculated based on the conversion table of equivalent effective dose ratios from humans to animals, considering body weight. We chose low, intermediate, and high doses corresponding to 1×, 2×, and 4× the recommended human intake, respectively. Mice were randomly distributed into five groups (12 mice per group): a sham group, an ovariectomy (OVX) group, a low-caffeine group (27.72 mg/kg caffeine, equivalent to approximately 200 mg daily intake for a 60-kg human), a medium caffeine group (55.44 mg/kg caffeine, equivalent to approximately 400 mg daily intake for a 60-kg human), and a high-caffeine group (110.88 mg/kg caffeine, equivalent to approximately 800 mg daily intake for a 60-kg human). Mice in the OVX group received normal saline, while mice in the low-, medium-, and high-dose caffeine groups received their designated caffeine doses. All mice were anesthetized with 5% chloral hydrate. In the sham group, the ovaries were simply exposed without disturbing the surrounding adipose tissue. Bilateral ovariectomy was performed in the OVX, low-caffeine, medium-caffeine, and high-caffeine groups. After a 1-week post-operative recovery period, mice in the OVX and low-, medium-, and high-caffeine groups received gavage with normal saline or their respective caffeine doses, respectively. Twelve weeks later, all groups were administered an overdose of chloral hydrate, followed by removal of bilateral femurs and blood samples for further study.

### 2.6 Microcomputed tomography

The femurs of mice were fixed in 4% paraformaldehyde (PFA). Micro-CT (SkyScan 1276, Bruker, Germany) was used to scan each distal femoral metaphysis. The analysis conditions met the following parameters: the voltage was 69 kV, the electric current was 362 μA, and the resolution was 0.5 mm. Then, the scans were integrated into 2D and 3D images. Quantitative data on femur parameters were obtained as follows: bone mineral density (BMD), bone volume/tissue volume (BV/TV), bone surface/tissue volume (BS/TV), trabecular bone number (Tb.N), and trabecular separation (Tb.Sp). Using built-in software, these parameters were calculated.

### 2.7 Histological analysis

Within 2 weeks, the femurs were embedded in paraffin, sectioned using a microtome, and then stained with hematoxylin and eosin (HE), the TRAP staining kit, and the ALP staining kit, respectively. An Olympus BX53 Light Microscope was used to observe and photograph the trabecular bone area of the femurs. The number of TRAP-positive multinucleated osteoclasts with three or more nuclei and ALP-positive osteoblasts was then quantified.

### 2.8 Serum biochemistry analysis

Blood was collected from one mouse eye for hormonal and biochemical analysis. After centrifugation at 1,000 *g* for 15 min, serum was collected. Following the manufacturer’s instructions, an ELISA Kit (Meimian, Jiangsu, China) was used to detect the levels of estrogen (E_2_), osteocalcin (OCN), acid phosphatase (ACP), alkaline phosphatase (ALP), calcium (Ca), and phosphorus (P) in the serum.

### 2.9 Statistical analysis

All data are presented as the mean ± standard error of the mean (SEM) of three to six independent experiments. Data were analyzed using one-way analysis of variance (ANOVA), followed by Duncan’s multiple range test. PRISM 8 statistical software (GraphPad Software, San Diego, CA) was used for the analyses. A *p*-value of less than 0.05 was considered statistically significant.

## 3 Results

### 3.1 Effects of caffeine on osteoclasts (RANKL-induced RAW 264.7 cells) and osteoblasts (MC3T3-E1 cells)

The cytotoxic effect of caffeine (0, 3.125, 6.25, 12.5, 25, 50, 100, 200, 400, and 800 μg/mL) on RANKL-induced RAW 264.7 cells and L-ascorbic acid + β-glycerophosphate-induced MC3T3-E1 cells was assessed using a CCK-8 assay. The CCK-8 assay results showed no significant cytotoxicity for caffeine doses below 50 μg/mL (Supplementary Figure S1).

**FIGURE 1:**
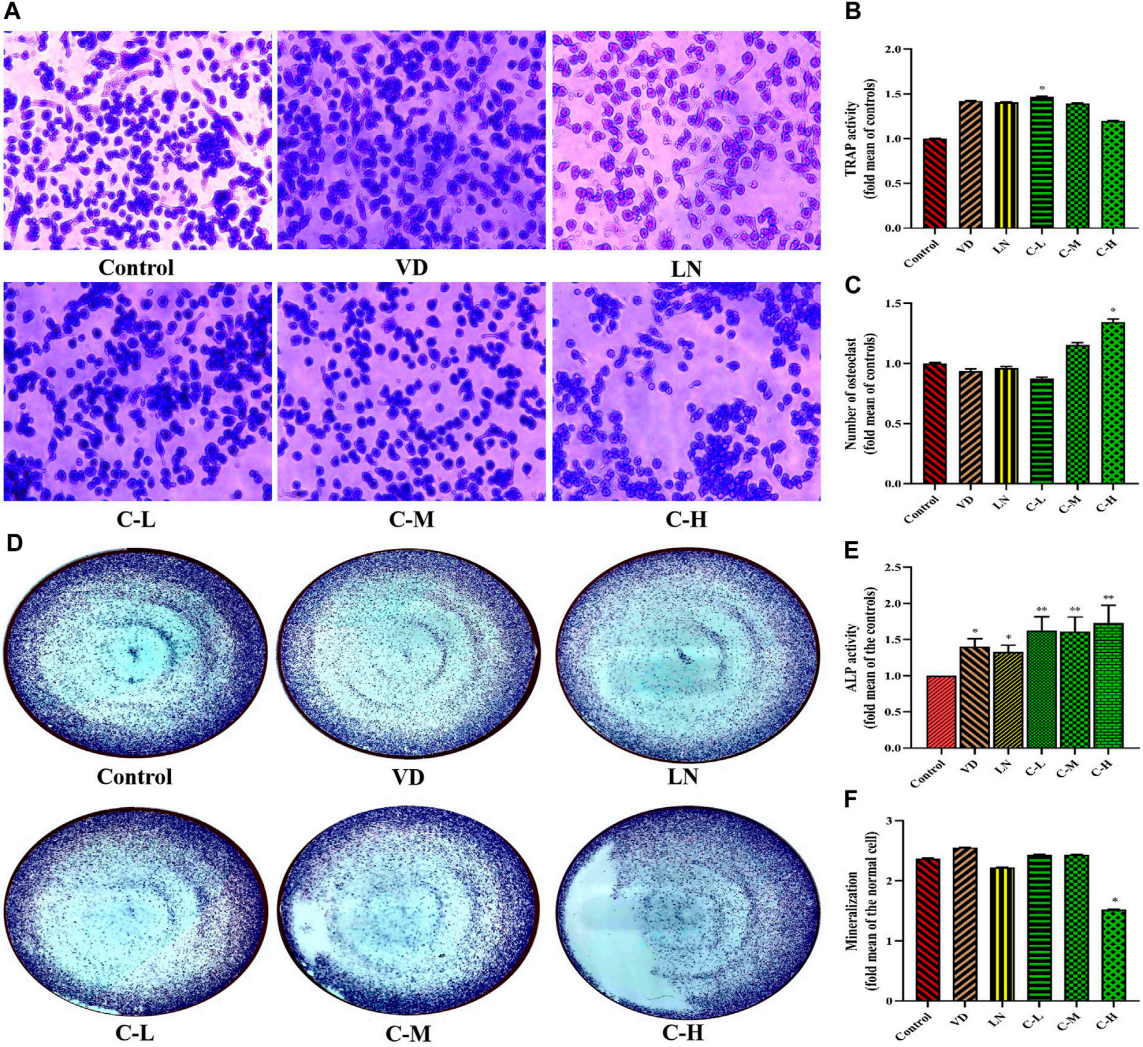
Effects of caffeine on osteoclasts and osteoblasts. CCK-8 analysis of caffeine’s cytotoxicity in RAW 264.7 cells at 24 h **(A)** and 48 h **(B)**. Cell proliferation of RANKL-induced RAW 264.7 cells after 5 days **(C)**. CCK-8 analysis of caffeine’s cytotoxicity in MC3T3-E1 cells at 24 h **(D)** and 48 h **(E)**. Cell proliferation of L-ascorbic acid + β-glycerophosphate-induced MC3T3-E1 cells after 14 days **(F)**. The experiments were repeated three times. **(A)** Formation of TRAP-positive cells from RANKL-induced RAW 264.7, treated with different concentrations of caffeine (3.125, 12.5, and 50 μg/mL) for 5 days; **(B)** activity of TRAP after 5 days of treatment; and **(C)** quantification of osteoclasts after 5 days of treatment. **(D)** Formation of ALP-positive cells from L-ascorbic acid + β-glycerophosphate-induced MC3T3-E1, treated with different doses of caffeine (3.125, 12.5, and 50 μg/mL) for 14 days; (E) activity of ALP after 14 days of treatment; and **(F)** quantification of mineralization in MC3T3-E1 cells treated with different levels of caffeine doses (3.125, 12.5, and 50 μg/mL) for 21 days. (*p < 0.05, **p < 0.01 vs. control group; CL: 3.125 μg/mL low-dose caffeine, CM: 12.5 μg/mL medium-dose caffeine, and CH: 50 μg/mL high-dose caffeine).

To further assess the effects of caffeine on osteoclast formation and function, a RANKL-induced osteoclastogenesis assay was performed in RAW 264.7 cells. The cells were exposed to various doses of caffeine (0, 3.125, 12.5, and 50 μg/mL). Multinucleated cells positive for TRAP activity were counted to quantify RANKL-induced osteoclast differentiation. Although caffeine treatment led to a decrease in TRAP activity (Figure 1B), there was a significant increase in the abundance of TRAP-positive multinucleated cells in the 50 μg/mL high-dose caffeine group compared to the control group (Figures 1A, C).

ALP activity is a well-established marker of osteogenic cell differentiation and is essential for bone formation (Mou et al., 2019). Bone mineralization is the process by which bones become hardened with calcium deposits, contributing to bone strength (Ramachandran et al., 2016). Therefore, investigating the effects of caffeine doses on bone formation is crucial. Data from Figures 1D, E show that MC3T3-E1 cells treated with caffeine (3.125, 12.5, and 50 μg/mL) exhibited a significant increase in ALP activity in both cell lysates and culture medium compared to the control group (*p* < 0.01). However, interestingly, cells in the high-dose caffeine group (50 μg/mL) did not show a corresponding increase in bone mineralization (Figure 1F).

Based on these results, it can be inferred that high doses of caffeine stimulate osteoclastogenesis while delaying osteoblastic mineralization. This suggests a disruption of bone homeostasis due to the imbalance between osteoclast and osteoblast activity.

### 3.2 Effects of different caffeine doses during RANKL-induced RAW 264.7 osteoclast differentiation on osteoclast metabolism

**FIGURE 2:**
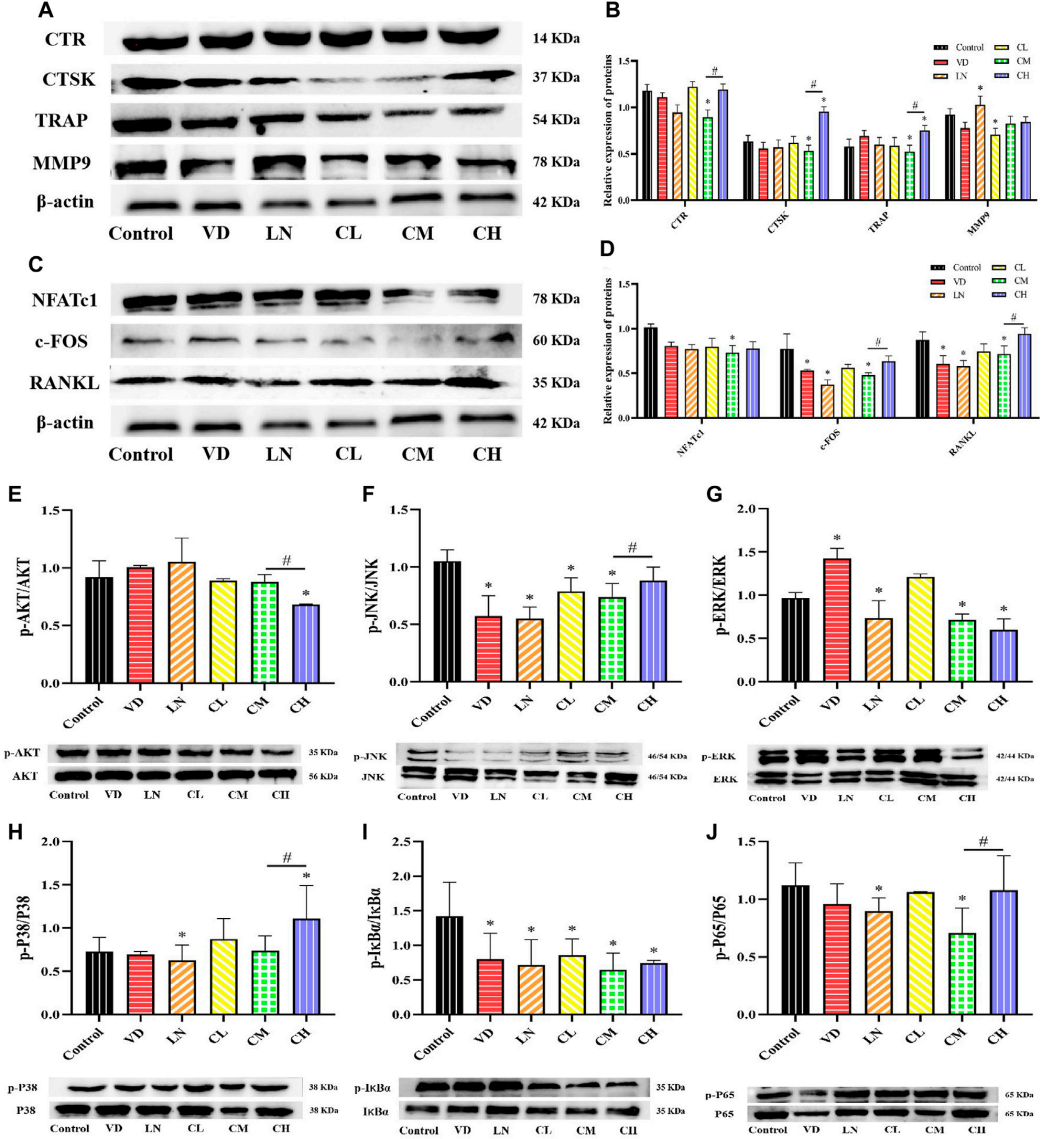
Effects of caffeine on several osteoclast-related genes and AKT, NF-κB, and MAPK signaling pathways. **(A, B)** Expression levels of MMP-9, CTSK, TRAP, and CTR in RANKL-induced RAW 264.7 cells. **(C, D)** Expression levels of NFATc1, c-Fos, RANKL, and RANKL-induced RAW 264.7 cells. **(E)** Representative images and quantification of the phosphorylation of AKT. **(F–H)** Representative images and quantification of the phosphorylation of the members of the NF-κB pathway (IκBɑ and P65). **(H–J)** Representative images and quantification of the phosphorylation of the members of MAPKs (ERK, JNK, and P38) (*p < 0.05 vs. control group; ^#^ *p* < 0.05 CM group vs. CH group; CL: 3.125 μg/mL low-dose caffeine, CM: 12.5 μg/mL medium-dose caffeine, and CH: 50 μg/mL high-dose caffeine).

Cathepsin K (CTSK), matrix metalloproteinase 9 (MMP9), calcitonin receptor (CTR), and TRAP are essential enzymes and receptors required for bone resorption during RANKL-induced osteoclastogenesis (Huang et al., 2021). As shown in Figures 2A, B, RAW 264.7 cells stimulated with RANKL and treated with caffeine exhibited a significant decrease in TRAP, CTR, and cathepsin K protein levels in the 12.5 μg/mL (medium dose) group compared to the control group (*p* < 0.05). However, the high-dose caffeine group expressed relatively higher protein levels of CTSK and TRAP than the control group, with a trend toward increased CTR and MMP9 protein levels. Therefore, the Western blot results suggest that caffeine affects osteoclast-related gene expression and differentiation/maturation. These findings indicate that the low and medium doses inhibited osteoclastogenesis, while a high dose promoted it.

A high level of RANKL is associated with osteoclast differentiation and activation. It strongly stimulates the major regulator of osteoclast differentiation, NFATc1 (Tang et al., 2019). Meanwhile, NFATc1 and c-Fos play crucial roles in osteoclastogenesis, with RANKL stimulating NFATc1 expression via c-Fos to promote osteoclastogenesis (Li et al., 2019). Our results showed that the protein expression levels of RANKL, c-Fos, and NFATc1 were not significantly affected by varying caffeine doses (Figures 2C, D). However, there was a significant decrease in the expression of RANKL, c-Fos, and NFATc1 in the 12.5 μg/mL caffeine group compared to the control group (*p* < 0.05). Interestingly, the high-dose caffeine group (50 μg/mL) showed a trend toward increased expression of these proteins, although not statistically significant. Therefore, a moderate dose of caffeine (12.5 μg/mL) may inhibit RANKL-mediated NFATc1 and c-Fos expression, thereby suppressing the expression of osteoclast marker genes and leading to the inhibition of osteoclastogenesis. Conversely, a high dose of caffeine may activate RANKL expression, potentially enhancing osteoclastogenesis.

The activation of the RANKL-stimulated AKT, mitogen-activated protein kinase (MAPKs), and NF-κB pathways is essential for osteoclastogenesis (Chen et al., 2020). To investigate the role of caffeine in these pathways, a Western blot assay was performed in RANKL-induced RAW 264.7 cells. The results revealed that AKT phosphorylation decreased with increasing caffeine doses (Figure 2E). For the MAPK pathways (ERK, JNK, and P38), the expressions of these downstream signaling molecules were significantly suppressed by 12.5 μg/mL (medium dose) treatment. Notably, P38 phosphorylation was significantly increased in the 50 μg/mL (high dose) caffeine group, and JNK phosphorylation also showed a trend toward an increase compared to the 12.5 μg/mL group, although not statistically significant (Figures 2F–H). The activation of the AKT and MAPK signaling pathways is crucial for osteoclast proliferation and differentiation, which ultimately affects bone resorption function (Kwak et al., 2020). Studies have shown that NF-κB inhibitors suppress RANKL-induced expression of c-Fos and NFATc1 by blocking NF-κB signaling (Quan et al., 2015). Western blot results showed that, following RANKL induction, the phosphorylation levels of NF-κB (IκBα and P65) were similarly attenuated by 12.5 μg/mL caffeine treatment. Conversely, the high-dose caffeine treatment (50 μg/mL) showed a trend toward increased phosphorylation levels (Figures 2I, J).

In conclusion, our results suggest that medium-dose caffeine (12.5 μg/mL) may exert inhibitory effects on the MAPKs and NF-κB pathways, leading to downregulation of osteoclastogenesis-related gene expression and subsequent suppression of osteoclast formation. Interestingly, a high dose of caffeine (50 μg/mL) promoted the phosphorylation of JNK, P38, and P65, which coincided with an increase in the expression of osteoclast-associated genes, potentially promoting osteoclastogenesis.

### 3.3 Effects of different caffeine doses during MC3T3-E1 osteoblast differentiation on osteoblast metabolism

**FIGURE 3:**
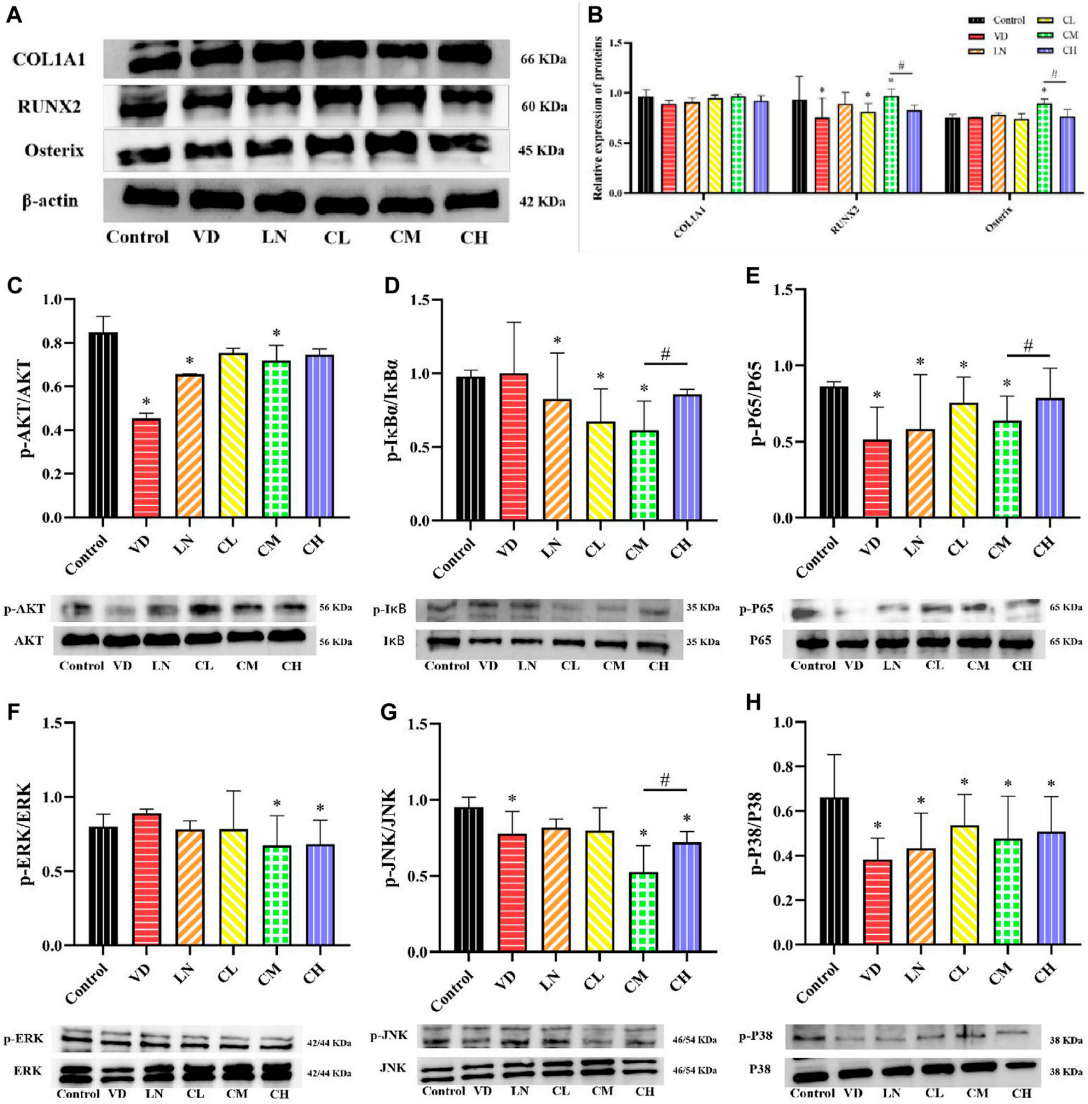
Effects of caffeine on several osteoblast-related genes and AKT, NF-κB, and MAPK signaling pathways. **(A, B)** Expression levels of COL1A1, Runx2, and Osterix in L-ascorbic acid + β-glycerophosphate-induced MC3T3-E1 cells. **(C)** Representative images and quantification of the phosphorylation of AKT. **(D, E)** Representative images and quantification of the phosphorylation of the members of the NF-κB pathway (IκBɑ and P65). **(F–H)** Representative images and quantification of the phosphorylation of the members of MAPKs (ERK, JNK, and P38) (*p < 0.05 vs. control group; ^#^ *p* < 0.05 CM group vs. CH group; CL: 3.125 μg/mL low-dose caffeine, CM: 12.5 μg/mL medium-dose caffeine, and CH: 50 μg/mL high-dose caffeine).

The differentiation of osteoblasts plays an important role in bone formation (Reddi et al., 2016). Runx2 is an osteogenic transcription factor that regulates the production of bone matrix proteins like ALP, type I collagen (COL1A1), osteopontin (OPN), and osteocalcin (OCN), influencing osteoblast differentiation (Ding et al., 2019; Xu et al., 2021). To investigate the role of caffeine in this process, we measured the protein levels of COL1A1, Runx2, and Osterix in MC3T3-E1 cells. We observed an increase in Runx2 and Osterix protein levels following treatment with medium-dose caffeine (12.5 μg/mL) compared to the control group (*p* < 0.05). However, the high-dose caffeine group (50 μg/mL) showed a trend toward decreased protein levels of COL1A1, Runx2, and Osterix compared to the medium-dose group (Figures 3A, B). Studies have shown that the AKT, NF-κB, and MAPK signaling pathways play a critical role in osteogenesis (Guo et al., 2018; Ren et al., 2019). To determine whether caffeine affects osteoblast-related gene expression by influencing the levels of AKT, NF-κB, and MAPK proteins, a Western blot analysis was performed. As shown in Figures 3C–H, the expressions of downstream signaling molecules in the AKT, NF-κB (IκBα, P65), and MAPK (ERK, JNK, and P38) pathways were significantly suppressed by the medium-dose caffeine treatment (12.5 μg/mL). Interestingly, high-dose caffeine treatment significantly increased the phosphorylation levels of NF-κB (IκBα, P65) and JNK, while AKT and P38 phosphorylation also showed a trend toward an increase compared to the medium-dose group, although not statistically significant.

In summary, our findings suggest that a medium dose of caffeine (12.5 μg/mL) could inhibit AKT, MAPK, and NF-κB pathways, potentially leading to the upregulation of osteogenesis through increased expression of osteoblast-related genes. Conversely, the high dose of caffeine (50 μg/mL) may activate AKT, MAPK, and NF-κB pathways, potentially downregulating osteogenesis by suppressing the expression of osteoblast-related genes.

### 3.4 Effects of caffeine on body weight and bone metabolism-related blood indicators in OVX mice

**FIGURE 4:**
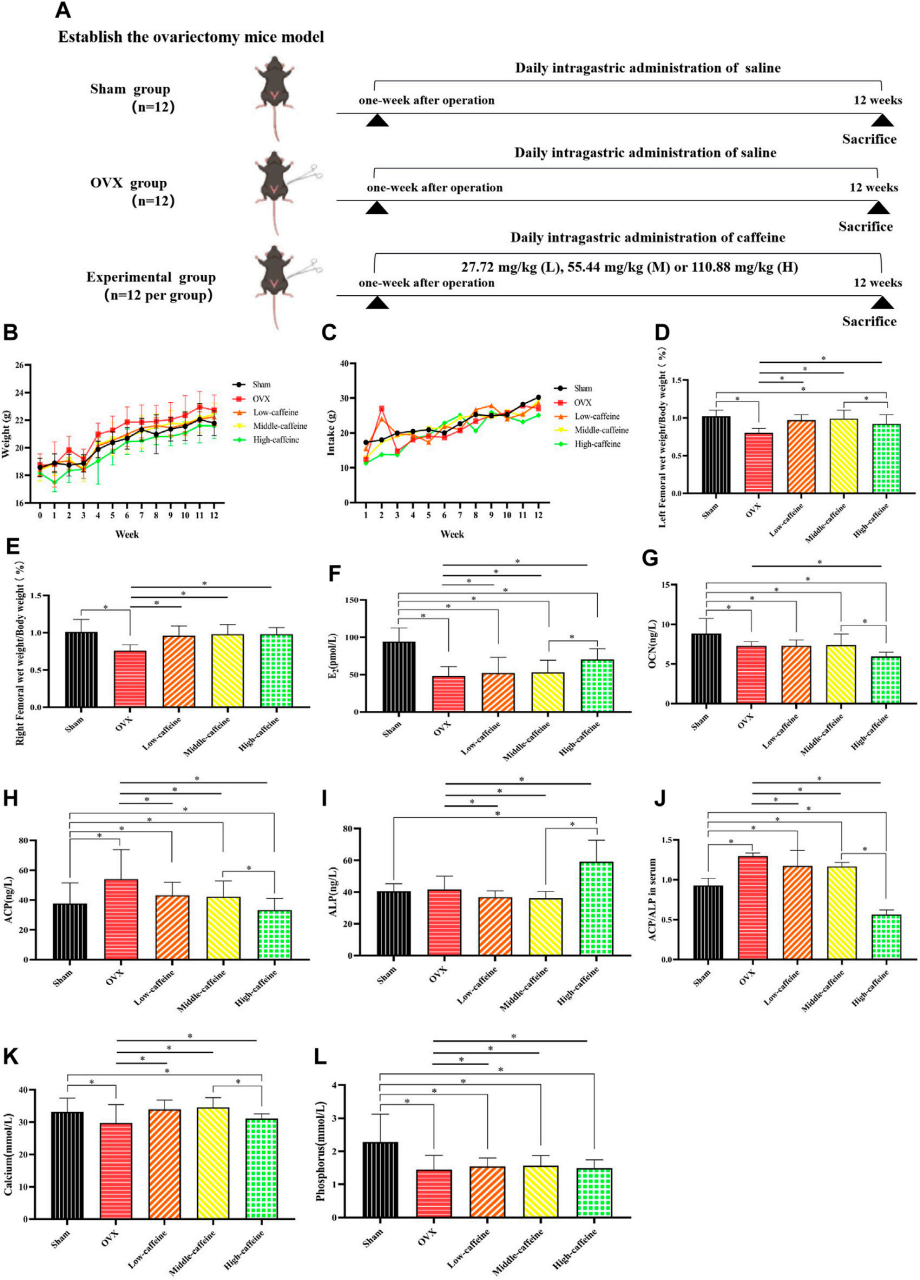
Effects of caffeine intake on body weight and bone metabolism-related blood indicators in OVX mice. **(A)** Study design; **(B)** effects of caffeine on the body weight of OVX mice; **(C)** effects of caffeine on the intake of OVX mice; **(D)** left femoral wet weight/body weight; **(E)** right femoral wet weight/body weight; **(F)** serum concentrations of E_2_; **(G)** serum concentrations of OCN; **(H)** serum concentrations of ACP; **(I)** serum concentrations of ALP; **(J)** ACP/ALP ratio; **(K)** serum concentrations of calcium; and **(L)** serum concentrations of phosphorus (*p < 0.05; low caffeine: 27.72 mg/kg, medium caffeine: 55.44 mg/kg, and high caffeine: 110.88 mg/kg).

First, we established an ovariectomy mouse model. Caffeine or saline was administered by gavage for 3 days following surgery (Figure 4A). Body weight is often used as a measure of health, growth, and development (Jiao et al., 2017). As shown in Figures 4B, C, treatment with caffeine at intragastric infusion doses of 27.72 mg/kg, 55.44 mg/kg, and 110.88 mg/kg did not significantly reduce OVX-induced weight loss, indicating that caffeine had no adverse effect on mouse growth. We further investigated whether caffeine could influence femur growth in OVX mice. The organ coefficients of the left and right wet femurs in the OVX group were significantly lower than those measured in the sham group (*p* < 0.05; Figures 4D, E). However, treatment with caffeine significantly increased the left and right femur weights in OVX mice compared to the OVX group (*p* < 0.05).

We further investigated the effects of caffeine on bone metabolism-related blood indicators in OVX mice. As expected, ovariectomy significantly decreased serum E_2_ levels compared to the sham group (*p* < 0.05, Figure 4F). Interestingly, only the high-dose caffeine group (110.88 mg/kg) showed a significant increase in E_2_ levels compared to the OVX group (*p* < 0.05). Additionally, we measured serum levels of biomarkers for bone formation (ALP and OCN) and bone resorption (ACP) (Figures 4G–I). In the OVX group, ACP levels were significantly higher than in the sham group (*p* < 0.05). Notably, the high-dose group also exhibited a significant decrease in OCN levels compared to the sham group (*p* < 0.05). However, treatment with caffeine at 110.88 mg/kg (high dose) reversed these trends, with decreased ACP levels (Figure 4H) and increased ALP levels (Figure 4I) compared to the OVX group (*p* < 0.05). Since ALP indicates osteogenesis and ACP reflects bone resorption, the ACP/ALP ratio can be used to assess the balance of bone remodeling (Hwang et al., 2016; Zhang et al., 2018). As expected, the ACP/ALP ratio in serum was significantly increased after OVX compared to the sham group (*p* < 0.05, Figure 4J). Importantly, all three caffeine treatment groups displayed lower ACP/ALP ratios compared to the OVX group (*p* < 0.01), suggesting a regulatory effect on bone remodeling. However, the calculated ACP/ALP ratio in the high-dose caffeine group was very low, which might indicate a disruption of bone homeostasis. Finally, we examined calcium and phosphorus ion levels, which are crucial for maintaining bone health. The analysis revealed that calcium levels were significantly decreased in both the OVX and high-dose caffeine groups compared to the sham group (*p* < 0.05, Figure 4K). Serum phosphorus levels in the OVX group and all caffeine treatment groups were also significantly lower than those in the sham group (*p* < 0.05; Figure 4L).

### 3.5 Caffeine regulates physiological bone-related indexes in ovariectomized mice

**FIGURE 5:**
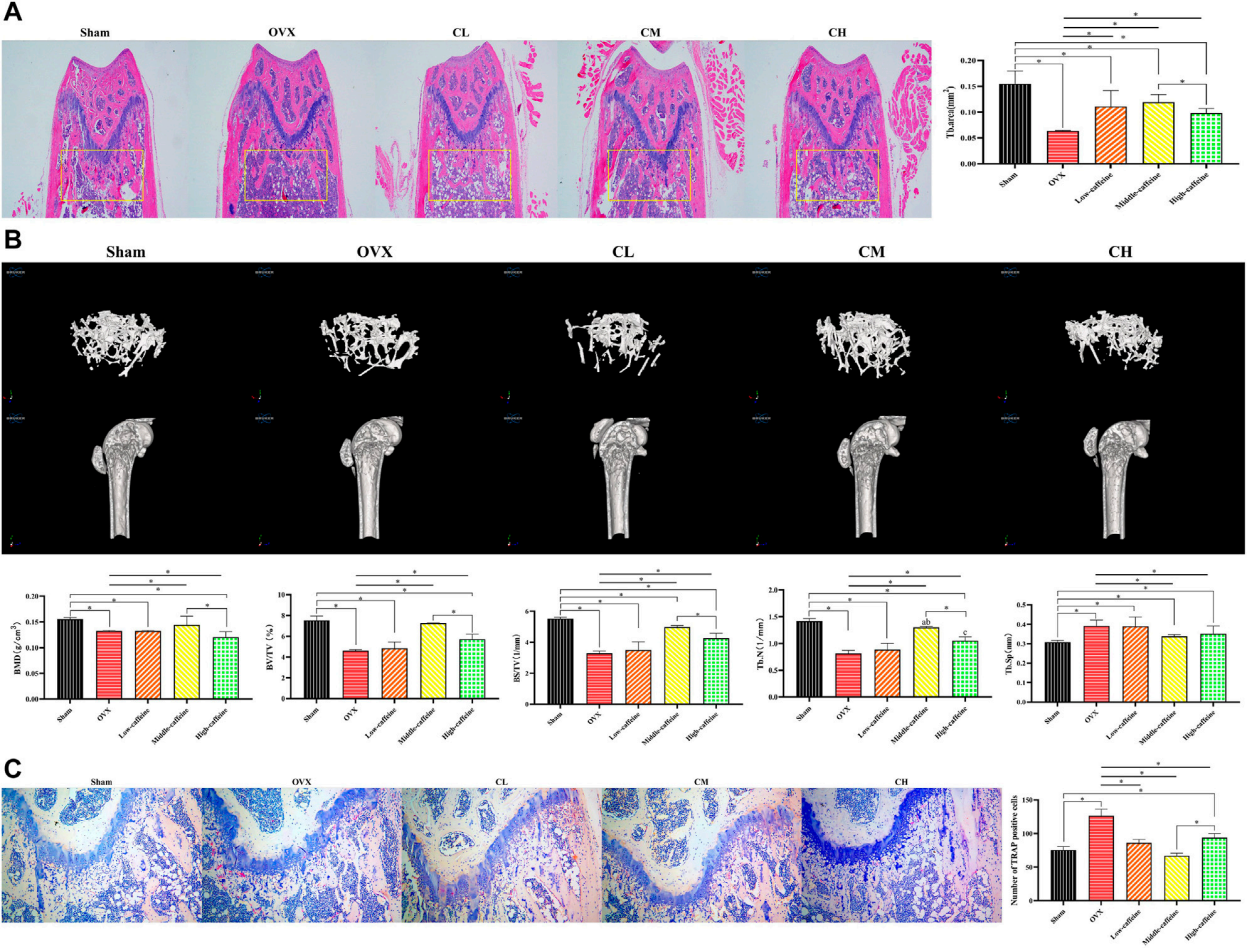
Caffeine attenuates ovariectomy-induced bone loss *in vivo*. **(A)** Representative H&E staining and quantification of the distal femoral trabecular area from each group 12 weeks after ovariectomy. **(B)** Micro-CT analysis of distal femurs from sham, OVX, and OVX + caffeine groups. Parameter quantification analysis: BMD, BV/TV, BS/TV, Tb.N, and Tb.Sp. **(C)** Representative images of TRAP-stained distal femoral sections and quantification of TRAP-positive cells from each group (*p < 0.05; low caffeine: 27.72 mg/kg, medium caffeine: 55.44 mg/kg, and high caffeine: 110.88 mg/kg).

To further assess the effectiveness of caffeine *in vivo*, we performed ovariectomy in mice to mimic the pathological bone loss that occurs after estrogen withdrawal. HE staining of femoral bone sections revealed a significant decrease in the trabecular bone area in the OVX group compared to the sham group (*p* < 0.05; Figure 5A). Caffeine treatment appeared to mitigate the trabecular bone loss caused by estrogen deficiency compared to the OVX group. Interestingly, long-term intake of a high dose (110.88 mg/kg) of caffeine may have caused some trabecular bone loss compared to the medium-dose caffeine intake.

To further evaluate bone formation, micro-CT analysis was performed to confirm the HE staining results. Micro-CT revealed a significant degree of trabecular bone loss in the OVX group compared to the sham group. Notably, the medium-dose caffeine treatment group showed preservation of bone mass compared to the OVX group, as evidenced by increased values of BMD, BV/TV, BS/TV, Tb.N, and Tb.Sp in the 2-dimensional and 3-dimensional micro-CT images (Figure 5B). These findings are consistent with the HE staining data. Additionally, long-term intake of high-dose (110.88 mg/kg) caffeine may have resulted in more trabecular bone loss compared to medium-dose caffeine intake.

Given that the period of osteoclastogenesis and bone resorption is shorter than the differentiation and maturation of osteoblasts required for bone matrix mineralization, bone formation lags behind bone resorption, resulting in a net decrease in bone mass (Karsdal et al., 2005). To investigate whether caffeine intervention inhibits osteoclasts, we performed TRAP staining. The results showed that distal femur sections from ovariectomized mice had an increased number of TRAP-positive multinucleated cells in the trabecular area compared to the sham group. Conversely, the caffeine group exhibited fewer osteoclasts compared to the OVX group (Figure 5C). Interestingly, long-term intake of high-dose (110.88 mg/kg) caffeine resulted in more TRAP-positive multinucleated cells in the trabecular area than medium-dose (55.44 mg/kg) caffeine intake.

In conclusion, our findings suggest that a moderate dose of caffeine (55.44 mg/kg) may attenuate OVX-associated bone loss by inhibiting osteoclast activity and promoting osteogenesis. Conversely, long-term intake of a high dose of caffeine (110.88 mg/kg) may disrupt osteogenesis and promote osteoclast activity, thereby disturbing bone homeostasis.

## 4 Discussion

**FIGURE 6:**
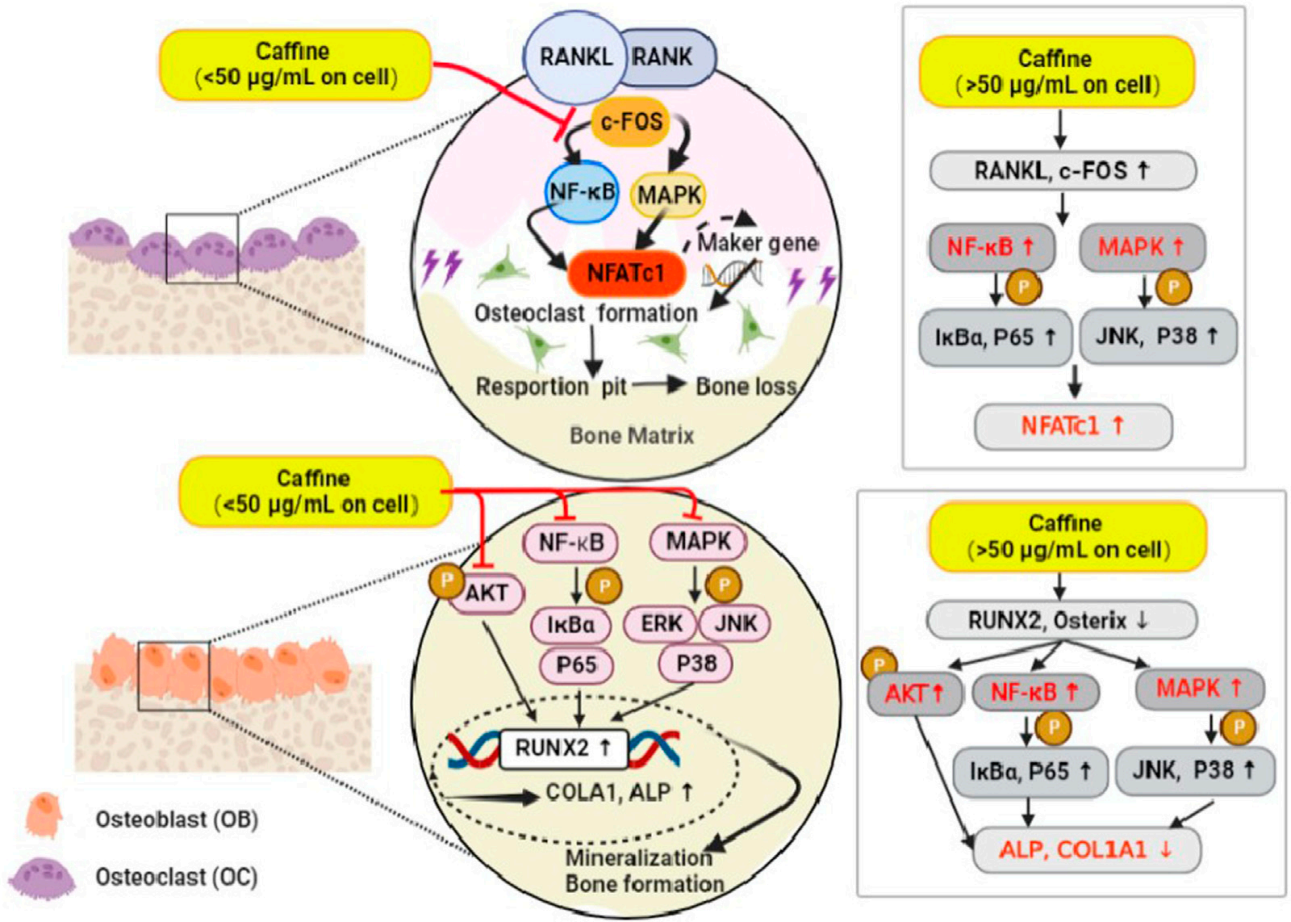
A brief diagram of the mechanism of caffeine inhibitory effects on RANKL-mediated osteoclastogenesis and osteoblastogenesis.

This study is the first to report the effects of caffeine on osteoclastogenesis and osteoblastogenesis, both *in vitro* and *in vivo*. *In vitro*, a moderate dose of caffeine (12.5 μg/mL) inhibited RANKL-induced signaling pathways (MAPKs and NF-κB) by blocking the interaction of RANK with c-Fos. Furthermore, it inhibited the MAPK, NF-κB, and AKT pathways that stimulate Runx2 expression, thereby promoting osteogenesis. *In vivo*, a moderate dose of caffeine (55.44 mg/kg) protected against ovariectomy-induced bone loss by inhibiting osteoclastogenesis and promoting osteogenesis. In contrast, a high dose of caffeine (50 μg/mL) enhanced RANKL signaling pathways (MAPKs and NF-κB) by facilitating the interaction between RANK and c-Fos. It also activated the phosphorylation of IκBα, P65, and JNK, which decreased Runx2 expression and thus suppressed osteogenesis. Animal experiments showed that the high-dose caffeine group (110.88 mg/kg) further exacerbated bone loss and reduced bone strength by inhibiting osteogenesis and promoting osteoclastogenesis (Figure 6).

Caffeine consumption has been linked to an increased risk of fractures in bones weakened by osteoporosis, as observed in mouse models (Kamagata-Kiyoura et al., 1999). Researchers have found that caffeine can stimulate the formation of osteoclasts, cells responsible for bone resorption, leading to lower bone mineral density (Lacerda et al., 2010). However, other studies suggest that moderate coffee consumption does not cause long-term negative health effects in healthy individuals (Poole et al., 2017), and consuming up to 400 mg of caffeine daily is reportedly safe (Doepker et al., 2018). The effects of caffeine on bone metabolism remain unclear, with some studies suggesting negative impacts and others finding no significant effects. Osteoclast activation disrupts the delicate balance between bone resorption and bone formation, impacting bone microstructure. This imbalance has been linked to various bone disorders, including postmenopausal osteoporosis (PMOP) (Reid, 2008). MC3T3-E1 cell cultures are a valuable tool for studying osteoblast cells responsible for bone formation. These cells secrete proteins essential for bone matrix and mineralization during growth and development (Cann et al., 1984). Notably, osteoblasts play a crucial role in both bone formation and resorption in osteoporosis (Fan et al., 2017). Therefore, a potential strategy for treating pathological bone loss involves regulating osteoclast and osteoblast activity. Inhibiting overactive osteoclastogenesis or promoting osteoblastogenesis could be an effective approach to finding a cure for these diseases.

This study investigated the potential inhibitory or stimulatory effects of caffeine on osteoclastogenesis, osteoblastogenesis, and the underlying molecular mechanisms. *In vitro* experiments revealed minimal cytotoxic effects on RAW 264.7 cells and MC3T3-E1 cells at doses below 50 μg/mL. Based on these findings, 3.125, 12.5, and 50 μg/mL doses were chosen for further studies. We observed that a moderate dose of caffeine (12.5 μg/mL) potently suppressed the differentiation of RAW 264.7 cells into mature osteoclasts induced by RANKL. Interestingly, the same dose (12.5 μg/mL) promoted osteoblast mineralization, suggesting a role in orchestrating the bone-building process.

Osteoclastogenesis occurs in two stages. First, M-CSF stimulates the survival, proliferation, and differentiation of bone marrow macrophages (BMMCs). Second, RANKL binds to and activates the RANK receptor, c-Fos is recruited, which in turn activates downstream signaling cascades like the NF-κB and MAPK pathways. The RANK-c-Fos interaction activates these pathways through phosphorylation of IκBα, P65, JNK, and P38. Finally, the activation of the NF-κB and MAPK pathways leads to the activation and translocation of NFATc1, the master transcription factor regulating genes associated with osteoclastogenesis, including TRAP, CTR, MMP-9, and CTSK. Our study demonstrated that a moderate dose of caffeine (12.5 μg/mL) could disrupt RANKL-induced c-Fos recruitment, thereby inhibiting both the NF-κB and MAPK pathways and subsequent NFATc1 activation and translocation. Consequently, TRAP, CTR, and CTSK markers were downregulated. In contrast, a high dose of caffeine (50 μg/mL) promoted osteoclastogenesis and function by mimicking the effects of RANKL on c-Fos recruitment, pathway activation, and NFATc1 translocation. These findings align with previous research by Chen and Whitford (1999), which showed that high doses of caffeine in rodents increased urinary calcium excretion and decreased bone mineral content, suggesting a link between high caffeine intake and bone loss.

Osteoblasts derived from osteoprogenitor cells secrete osteoid, which increases bone mineral content and inhibits osteoclast activity. Bone morphogenetic proteins (BMPs) play a major role in osteoblast differentiation by promoting the expression of osteoblast-specific matrix proteins (Sykaras and Opperman, 2003; Chen et al., 2012). Upon BMP stimulation, Smad translocates into the nucleus and regulates the expression of several target genes, including Runx2 and Osterix (Nakashima et al., 2002; Bruderer et al., 2014). The osteoblast differentiation phenotype is characterized by high levels of ALP activity. ALP provides essential phosphate for osteoblast mineralization and the hydrolysis of inorganic pyrophosphate, an inhibitor of calcification (Lee et al., 2021). Additionally, differentiated osteoblasts produce collagen (COLA1), which further contributes to bone mineralization by facilitating calcium deposition (Banerjee et al., 2002). Our study found that a moderate dose of caffeine (12.5 μg/mL) increased ALP activity and calcium deposition, suggesting that it promotes osteoblast differentiation. Interestingly, this dose also disrupted the NF-κB, MAPK, and AKT pathways. Consequently, Runx2 and Osterix were fully activated. Furthermore, ALP and COLA1 expression markers were upregulated following treatment with a moderate dose of caffeine (12.5 μg/mL). In summary, a moderate dose of caffeine (12.5 μg/mL) protects MC3T3-E1 osteoblastic cells by enhancing osteoblast differentiation and inhibiting the phosphorylation of AKT, IκBα, P65, ERK, JNK, and P38. In contrast, a high dose of caffeine (50 μg/mL) yielded contrasting effects, promoting osteoblastogenesis and function through the activation of the AKT, NF-κB, and MAPK pathways while decreasing Runx2 and Osterix translocation.

Dysregulated bone resorption and bone formation are the main causes of PMOP pathogenesis. Estrogen withdrawal is associated with increased osteoclastogenesis, leading to enhanced bone resorption and substantial bone loss (Mundy, 2007). To mimic the pathological state of PMOP, we used ovariectomized mice as a model. HE staining and microcomputed tomography of the distal femurs revealed that the medium-dose caffeine group (55.44 mg/kg) significantly rescued bone loss after ovariectomy. TRAP staining showed a drastic decrease in the number of mature osteoclasts (TRAP-positive) around the trabecula. However, long-term intake of the high caffeine dose (110.88 mg/kg) may be associated with an increased risk of osteoporosis.

The majority of clinical studies indicate that daily caffeine consumption of up to 400 mg poses no significant health risks (Reuter et al., 2021b). However, caffeine is thought to negatively affect calcium homeostasis, potentially impacting bone health (Pu et al., 2016). *In vitro* studies have shown that caffeine inhibits osteoblast differentiation while enhancing osteoclast differentiation and maturation (Lu et al., 2008; Zhou et al., 2010). Consistent with these findings, our *in vivo* study discovered that a moderate dose of caffeine (55.44 mg/kg) also promoted osteogenesis. This is evidenced by a significant decrease in ACP activity in ovariectomized mice after treatment. Under these circumstances, overactivated osteoclastogenesis cannot be matched by an equivalent level of bone formation by osteoblasts within a single remodeling cycle, leading to a net loss of bone (Xiao et al., 2016). Interestingly, moderate-dose caffeine (55.44 mg/kg) reversed bone loss induced by ovariectomy and corrected the imbalance in bone coupling disorders by simultaneously inhibiting osteoclastogenesis and promoting osteogenesis. However, future clarification of caffeine’s molecular targets in osteoclastogenesis and osteoblastogenesis is necessary.

The adenosine receptors are widely distributed in the bone marrow (He and Cronstein, 2011). BMMs express the A_1_ receptor during osteoclast differentiation (Kara et al., 2010). Adenosine receptors also regulate the proliferation of osteoblasts and the secretion of cytokines from osteoblasts (Evans et al., 2006). Adenosine and adenosine receptors may play an important role in bone formation and remodeling. MAPKs and serine–threonine-specific kinases are affected by adenosine receptors by regulating intracellular cAMP levels (Verzijl and IJzerman, 2011). In osteoclasts, A_2A_R stimulation reduces the activation of ERK1/2 (Saulnier et al., 2012), and the results are in good agreement with those reported in the paper. Maia et al. (2020) found that the long-term administration of low doses of caffeine (10 mg/kg) was sufficient to recover alveolar bone damage in female rats. In addition, it shows that such a bone-positive effect was not generated by the blockade of the A_2A_ receptor pathway. Although studies have found that A_2A_R plays a role in caffeine metabolism (Nehlig et al., 1992), this is generally attributed to the antagonism of A_1_ and A_2A_ adenosine receptors and the resulting blockade of adenosine’s inhibitory action (Fredholm et al., 1999). When caffeine is consumed in moderate amounts, it acts by antagonizing adenosine receptors to protect against periodontal bone loss in adult males (Ng et al., 2014). These studies remain interesting and also provide some new ideas for our follow-up research. One key question is whether caffeine could regulate adenosine levels to stimulate bone homeostasis.

In conclusion, our study investigated the effects of caffeine on bone health. We found that a moderate dose of caffeine (55.44 mg/kg), equivalent to 4–5 cups of 200 mL of coffee per day in humans, acts as both an inhibitor of osteoclastogenesis and an inducer of osteogenesis, potentially offering a therapeutic approach for PMOP. This suggests caffeine could be a promising candidate for treating osteoporosis and other osteolytic bone diseases. However, higher doses exceeding 110.88 mg/kg (equivalent to 8–10 cups of 200 mL of coffee per day), may lead to significant health risks for bone homeostasis.

## Data Availability

The original contributions presented in the study are included in the article/Supplementary Material; further inquiries can be directed to the corresponding authors.
