# Supplementary material for: Caffeine regulates both osteoclast and osteoblast differentiation via the AKT, NF-κB, and MAPK pathways
Source: Front Pharmacol. 2024 Jun 13;15:1405173. doi: 10.3389/fphar.2024.1405173 (PMC11208461; doi:10.3389/fphar.2024.1405173)
Supplement: Supplementary file 1 [file Presentation1.PPTX]

## Slide 1
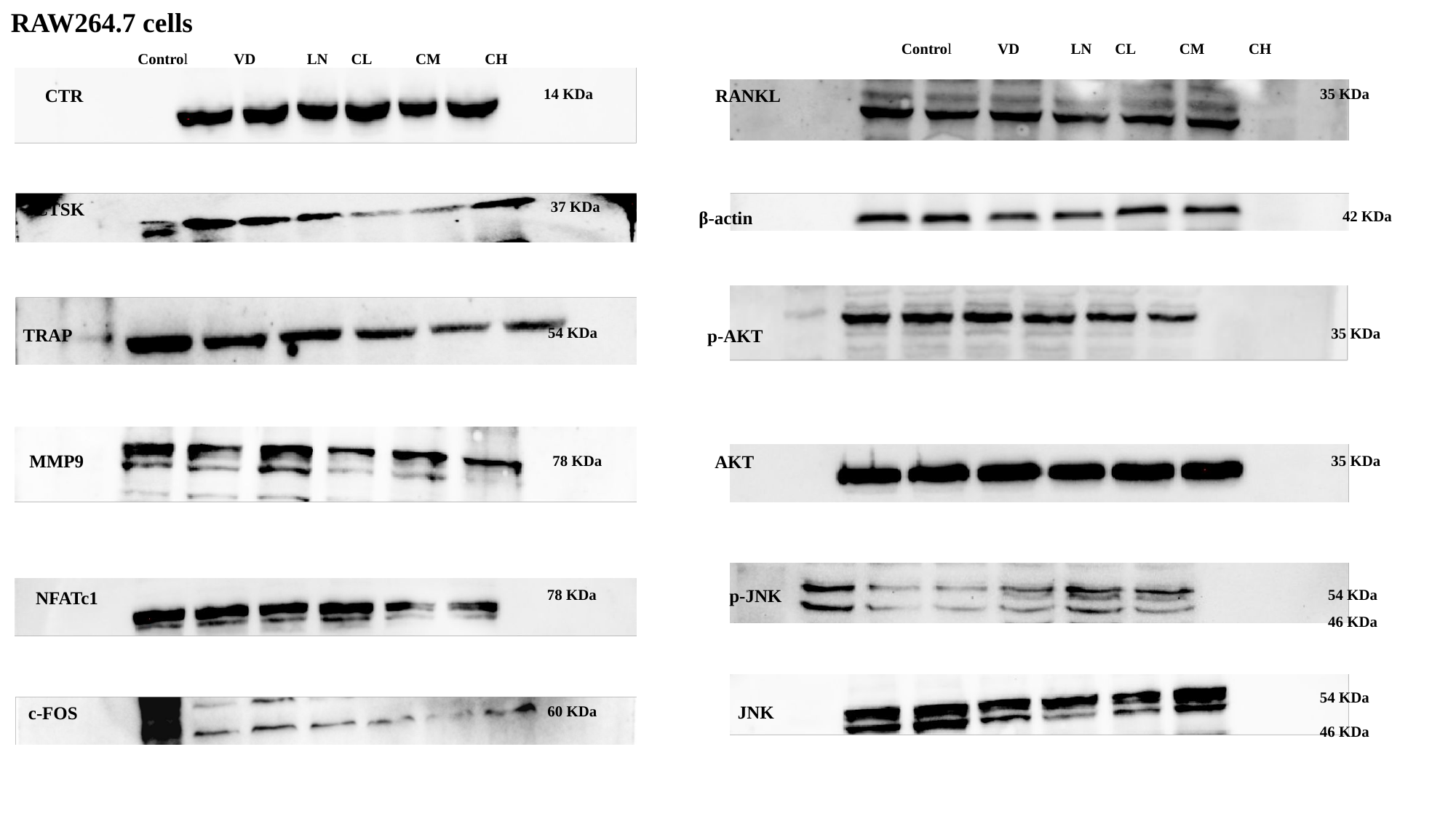

RAW264.7 cells
Control
VD
LN
CL
CM
CH
Control
VD
LN
CL
CM
CH
RANKL
35 KDa
CTR
14 KDa
37 KDa
CTSK
β-actin
42 KDa
54 KDa
TRAP
35 KDa
p-AKT
MMP9
78 KDa
AKT
35 KDa
p-JNK
78 KDa
NFATc1
54 KDa
46 KDa
54 KDa
46 KDa
JNK
c-FOS
60 KDa

## Slide 2
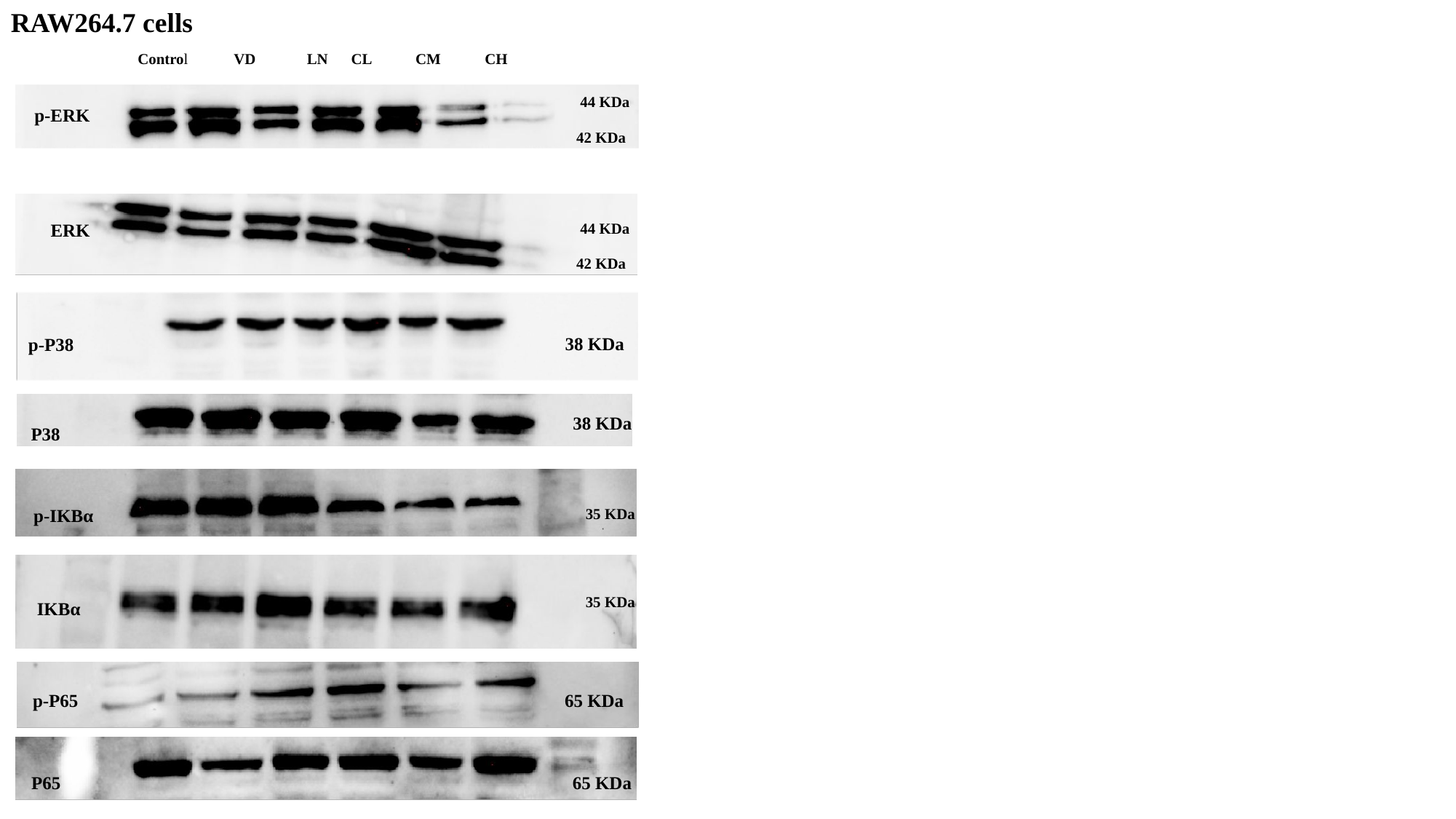

RAW264.7 cells
Control
VD
LN
CL
CM
CH
44 KDa
42 KDa
p-ERK
ERK
44 KDa
42 KDa
38 KDa
p-P38
38 KDa
P38
p-IKBα
35 KDa
35 KDa
IKBα
p-P65
65 KDa
P65
65 KDa

## Slide 3
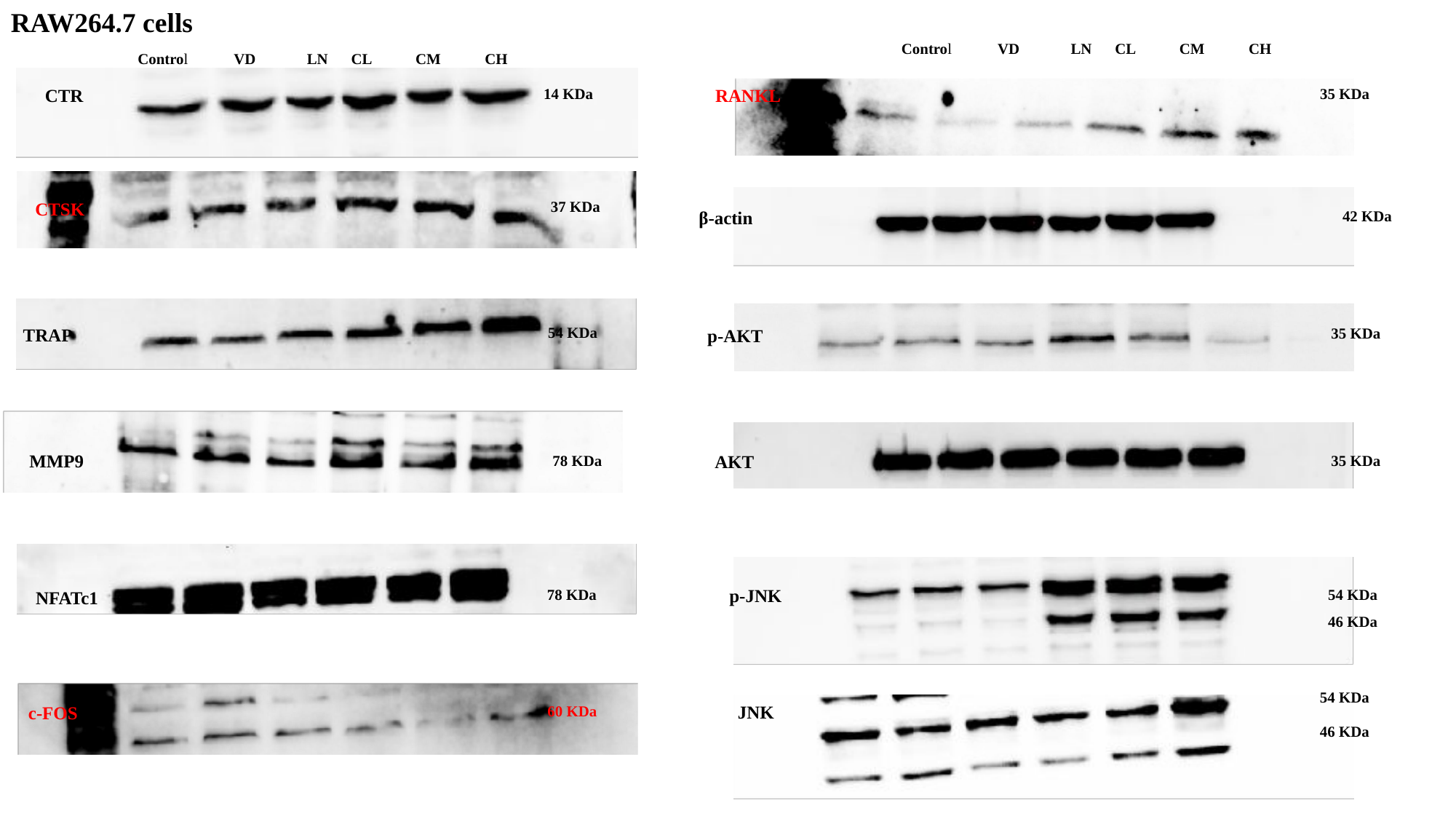

RAW264.7 cells
Control
VD
LN
CL
CM
CH
Control
VD
LN
CL
CM
CH
RANKL
35 KDa
CTR
14 KDa
37 KDa
CTSK
β-actin
42 KDa
54 KDa
TRAP
35 KDa
p-AKT
MMP9
78 KDa
AKT
35 KDa
p-JNK
78 KDa
NFATc1
54 KDa
46 KDa
54 KDa
46 KDa
JNK
c-FOS
60 KDa

## Slide 4
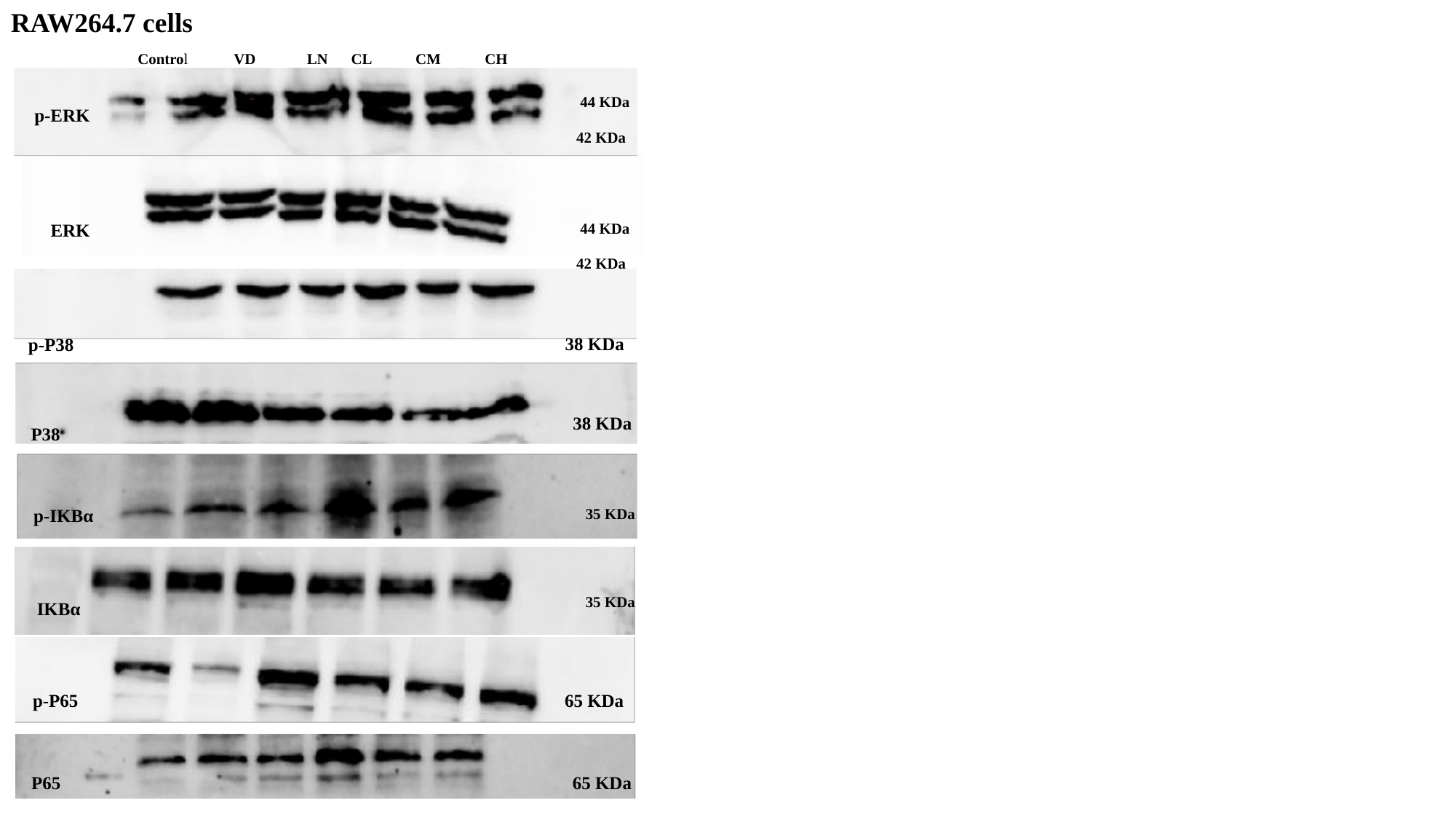

RAW264.7 cells
Control
VD
LN
CL
CM
CH
44 KDa
42 KDa
p-ERK
ERK
44 KDa
42 KDa
38 KDa
p-P38
38 KDa
P38
p-IKBα
35 KDa
35 KDa
IKBα
p-P65
65 KDa
P65
65 KDa

## Slide 5
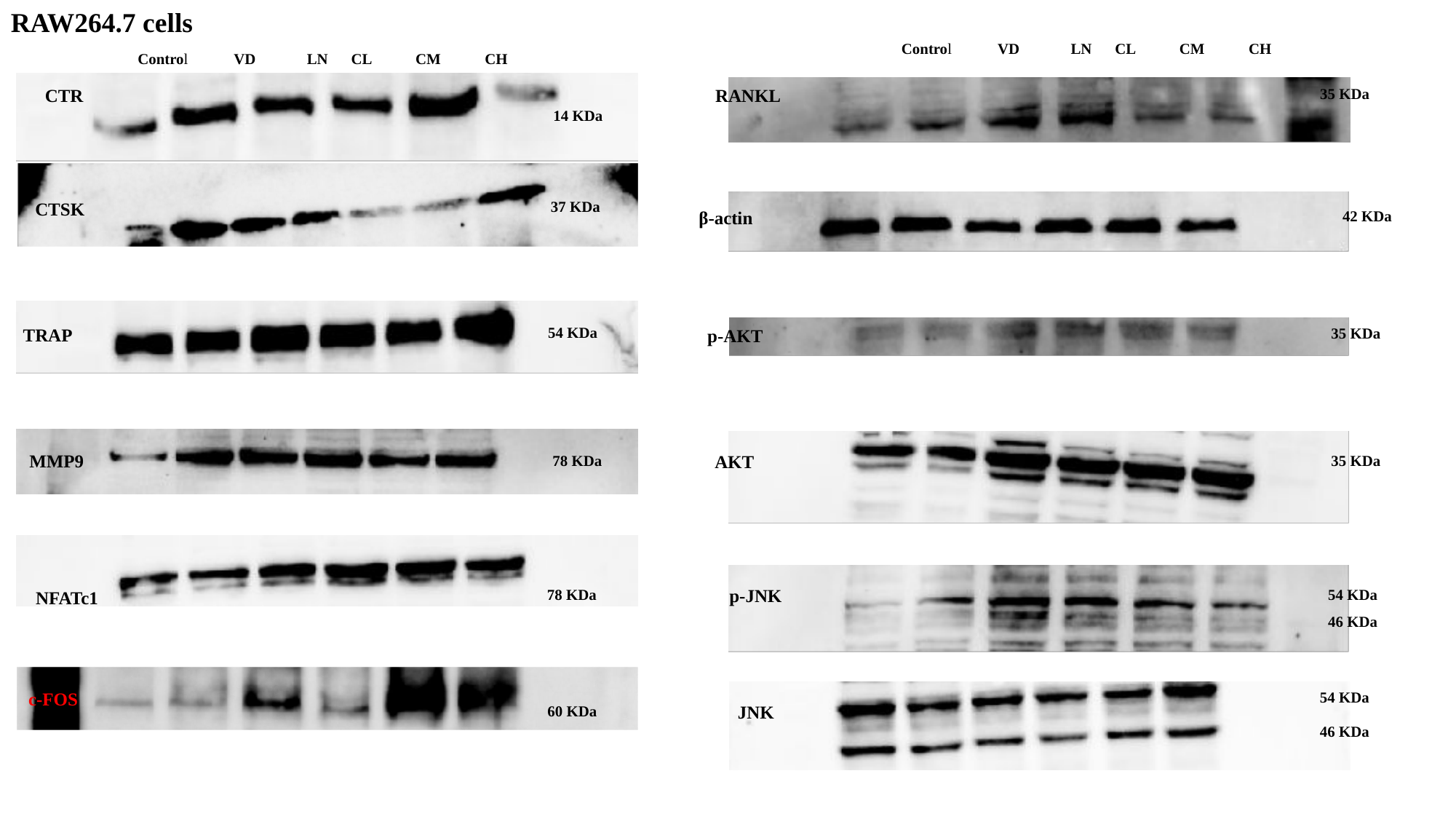

RAW264.7 cells
Control
VD
LN
CL
CM
CH
Control
VD
LN
CL
CM
CH
RANKL
35 KDa
CTR
14 KDa
37 KDa
CTSK
β-actin
42 KDa
54 KDa
TRAP
35 KDa
p-AKT
MMP9
78 KDa
AKT
35 KDa
p-JNK
78 KDa
NFATc1
54 KDa
46 KDa
c-FOS
60 KDa
54 KDa
46 KDa
JNK

## Slide 6
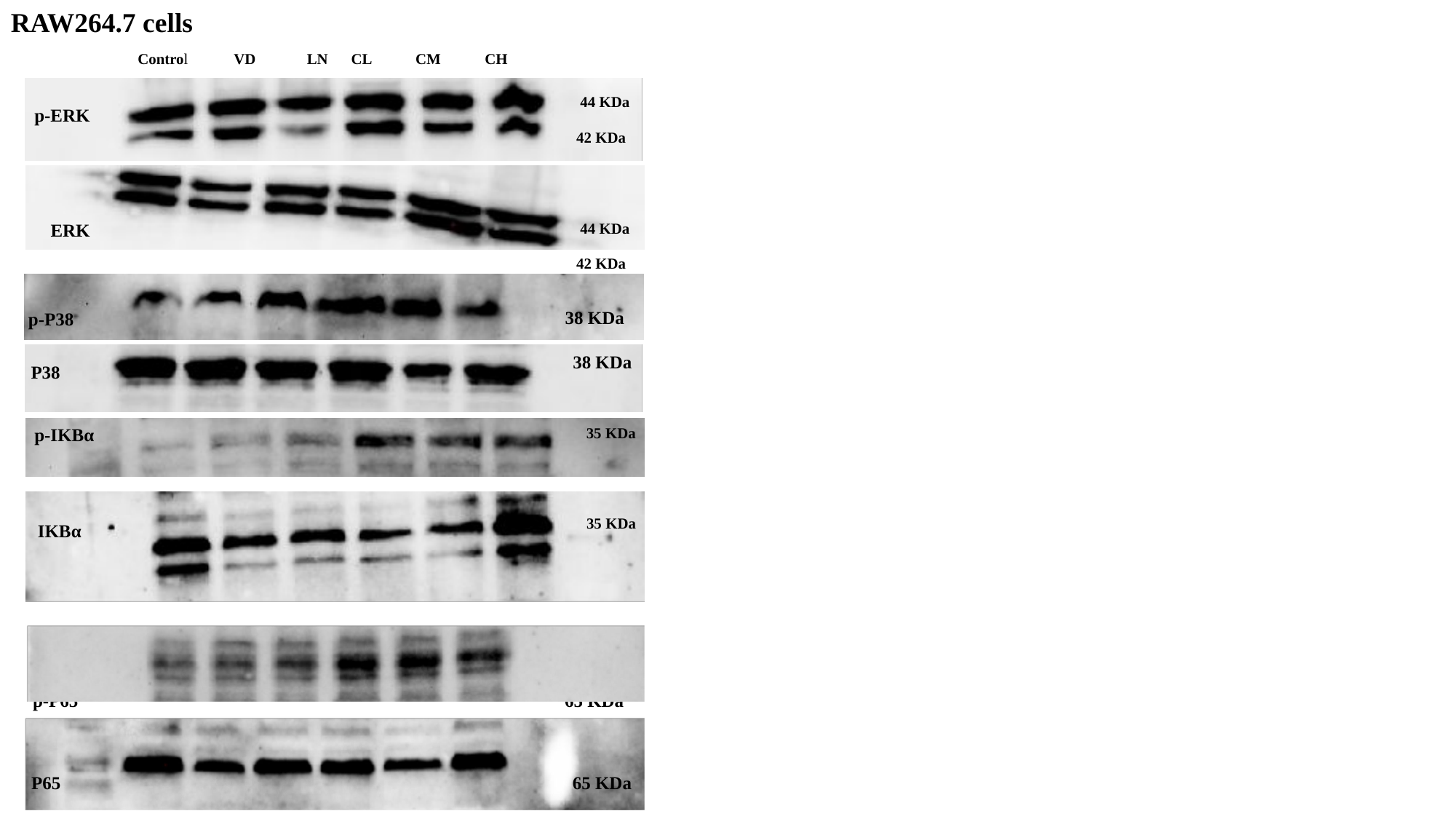

RAW264.7 cells
Control
VD
LN
CL
CM
CH
44 KDa
42 KDa
p-ERK
ERK
44 KDa
42 KDa
38 KDa
p-P38
38 KDa
P38
p-IKBα
35 KDa
35 KDa
IKBα
p-P65
65 KDa
P65
65 KDa

## Slide 7
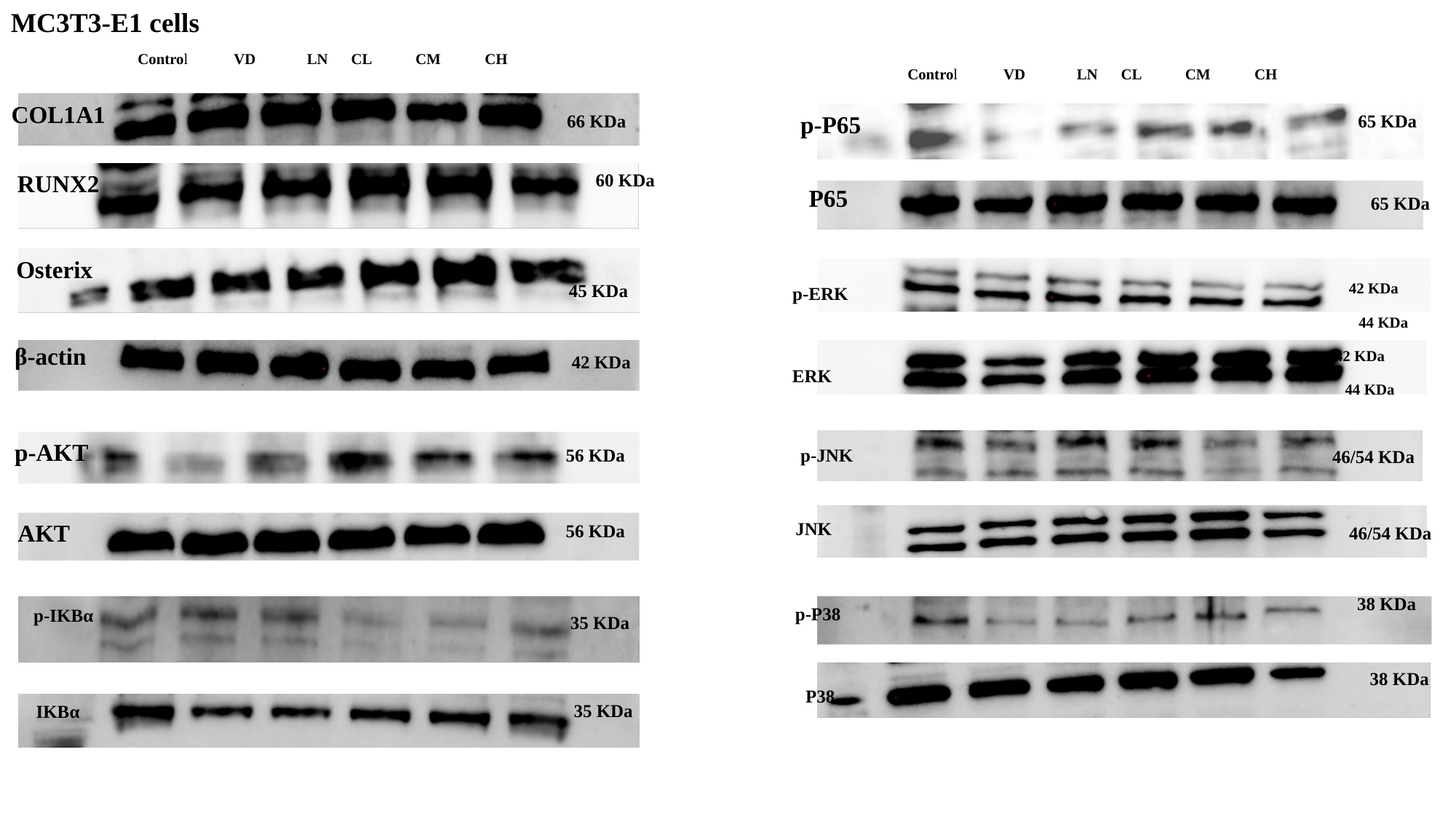

MC3T3-E1 cells
Control
VD
LN
CL
CM
CH
Control
VD
LN
CL
CM
CH
COL1A1
66 KDa
p-P65
65 KDa
RUNX2
60 KDa
P65
65 KDa
Osterix
45 KDa
42 KDa
p-ERK
44 KDa
β-actin
42 KDa
42 KDa
ERK
44 KDa
p-AKT
56 KDa
p-JNK
46/54 KDa
JNK
AKT
56 KDa
46/54 KDa
38 KDa
p-P38
p-IKBα
35 KDa
38 KDa
P38
35 KDa
IKBα

## Slide 8
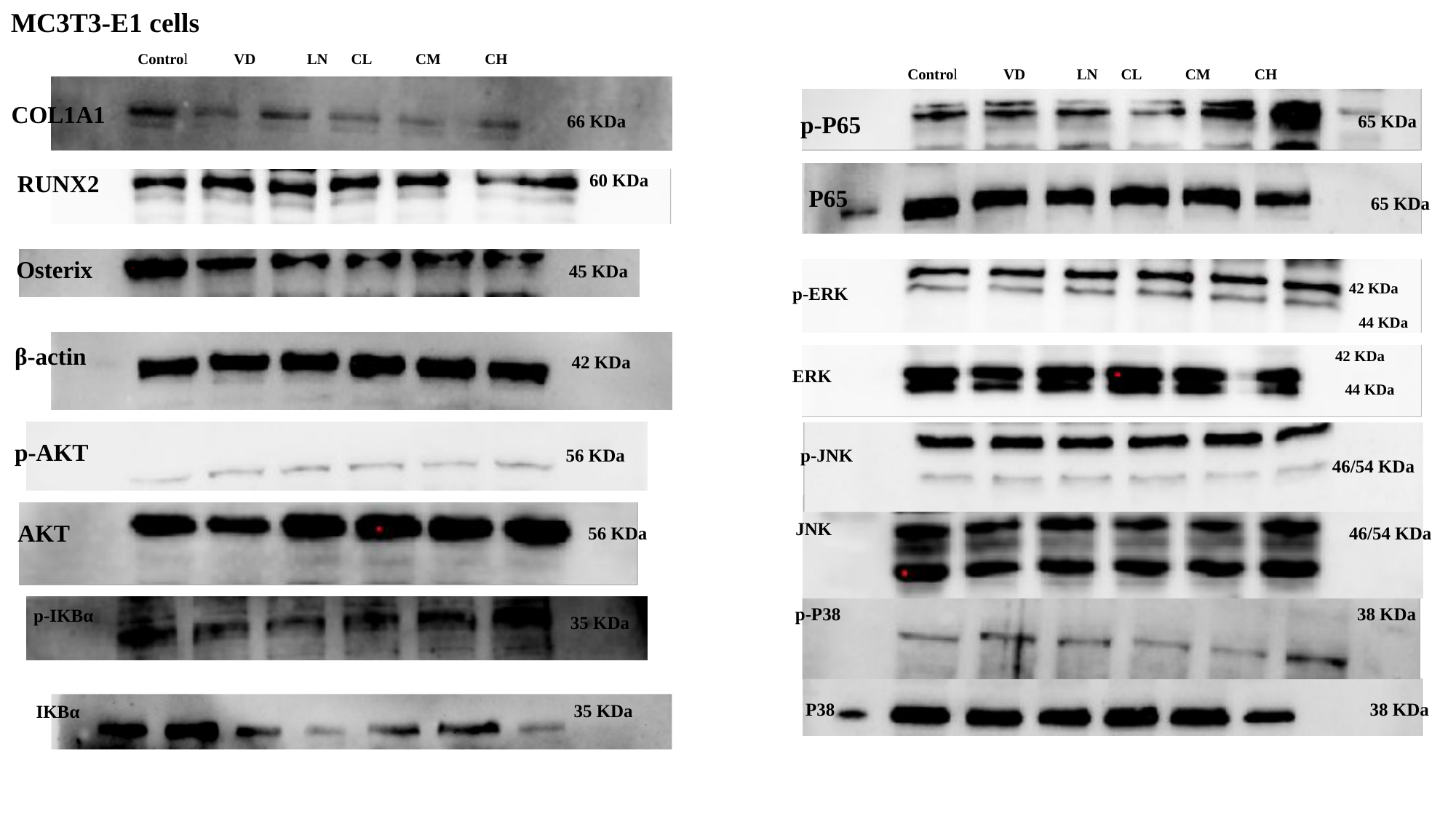

MC3T3-E1 cells
Control
VD
LN
CL
CM
CH
Control
VD
LN
CL
CM
CH
COL1A1
66 KDa
p-P65
65 KDa
RUNX2
60 KDa
P65
65 KDa
Osterix
45 KDa
42 KDa
p-ERK
44 KDa
β-actin
42 KDa
42 KDa
ERK
44 KDa
p-AKT
56 KDa
p-JNK
46/54 KDa
JNK
AKT
56 KDa
46/54 KDa
p-P38
38 KDa
p-IKBα
35 KDa
P38
38 KDa
35 KDa
IKBα

## Slide 9
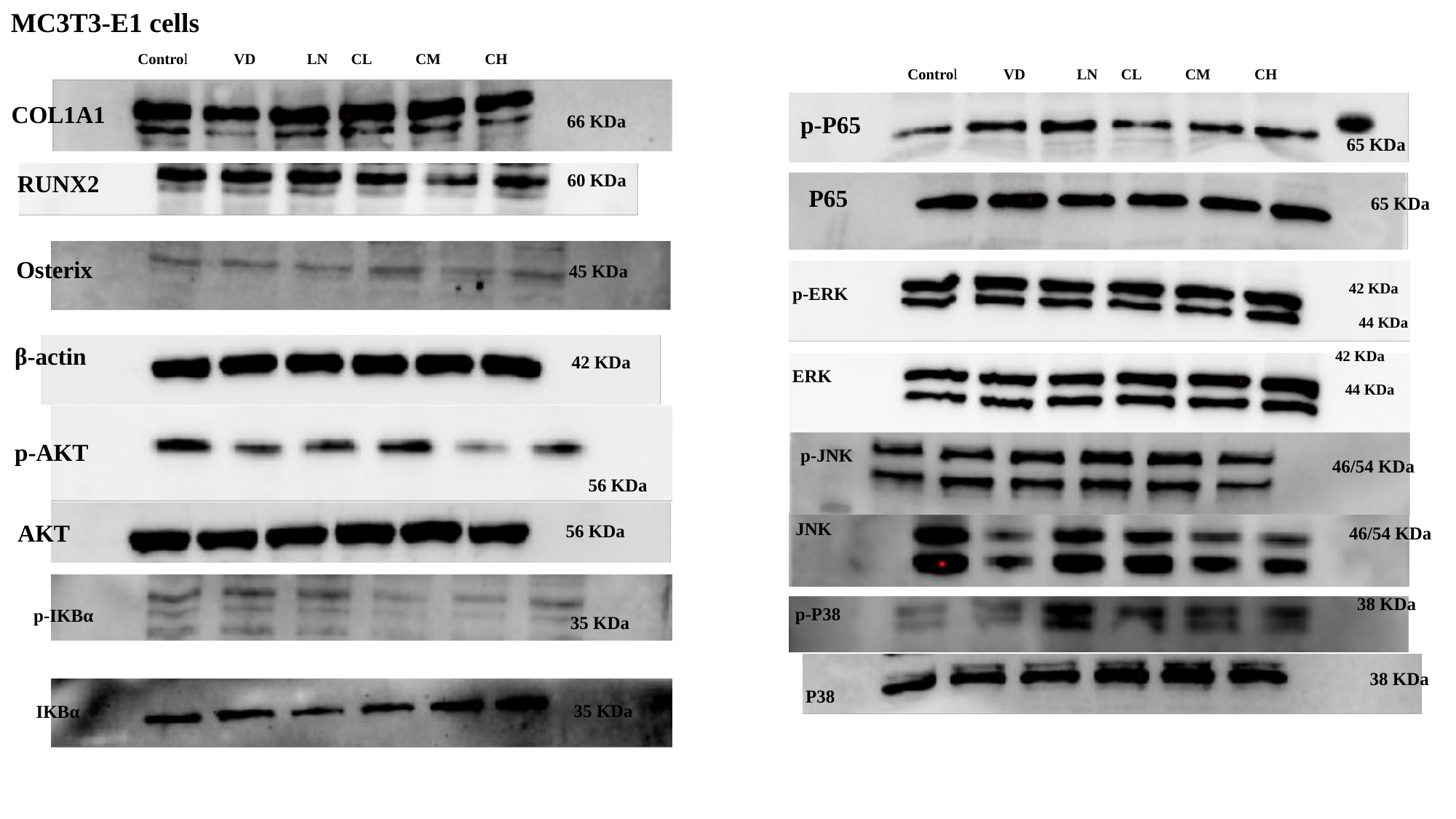

MC3T3-E1 cells
Control
VD
LN
CL
CM
CH
Control
VD
LN
CL
CM
CH
COL1A1
66 KDa
p-P65
65 KDa
RUNX2
60 KDa
P65
65 KDa
Osterix
45 KDa
42 KDa
p-ERK
44 KDa
β-actin
42 KDa
42 KDa
ERK
44 KDa
p-AKT
p-JNK
46/54 KDa
56 KDa
JNK
AKT
56 KDa
46/54 KDa
38 KDa
p-P38
p-IKBα
35 KDa
38 KDa
P38
35 KDa
IKBα
