# Supplementary material for: Caffeine regulates both osteoclast and osteoblast differentiation via the AKT, NF-κB, and MAPK pathways
Source: Front Pharmacol. 2024 Jun 13;15:1405173. doi: 10.3389/fphar.2024.1405173 (PMC11208461; doi:10.3389/fphar.2024.1405173)
Supplement: Supplementary file 2 [file DataSheet1.docx]

**Supplementary Information**

**Caffeine regulate bone homeostasis by modulating both osteoclast and osteoblast differentiation via the AKT, NF-**κ**B and MAPK pathways**

Yue Miao^a^, Lei Zhao^d^, Shuwen Lei^a^, Chunyan Zhao^a^, Qiuping Wang^a^, Chao Tan^a^, Chunxiu Peng^c,*^, Jiashun Gong^a,b,*^

^a^ College of Food Science and Technology, Yunnan Agricultural University, Kunming, Yunnan 650201, China

^b^ Yunnan Academy of Agricultural Science

^c^ College of Horticulture and Landscape, Yunnan Agricultural University, Kunming, 650201,China

^d^ College of Science, Yunnan Agricultural University, Kunming, 650201,China

Email addresses: [my12239406@163.com](mailto:my12239406@163.com) (Y. Miao), [zhaolei928@126.com](mailto:zhaolei928@126.com) (L. Zhao), [610252271@qq.com](mailto:610252271@qq.com) (S.W. Lei), [2052896274@qq.com](mailto:2052896274@qq.com) (C.Y. Zhao), soffywang@163.com (Q.P. Wang), [13208856843@163.com](mailto:13208856843@163.com) (C. Tan), 1045418854@qq.com (C.X. Peng), gong199@163.com (J.S. Gong)

* Corresponding author: Chunxiu Peng, e-mail: [1045418854@qq.com,](mailto:1045418854@qq.com,) Tel.: +86 13312524346; fax: +0086-871-65215534; Jiashun Gong, e-mail: gong199@163.com, Tel.: +86 13099910188; fax: +0086-871-65215534.

**
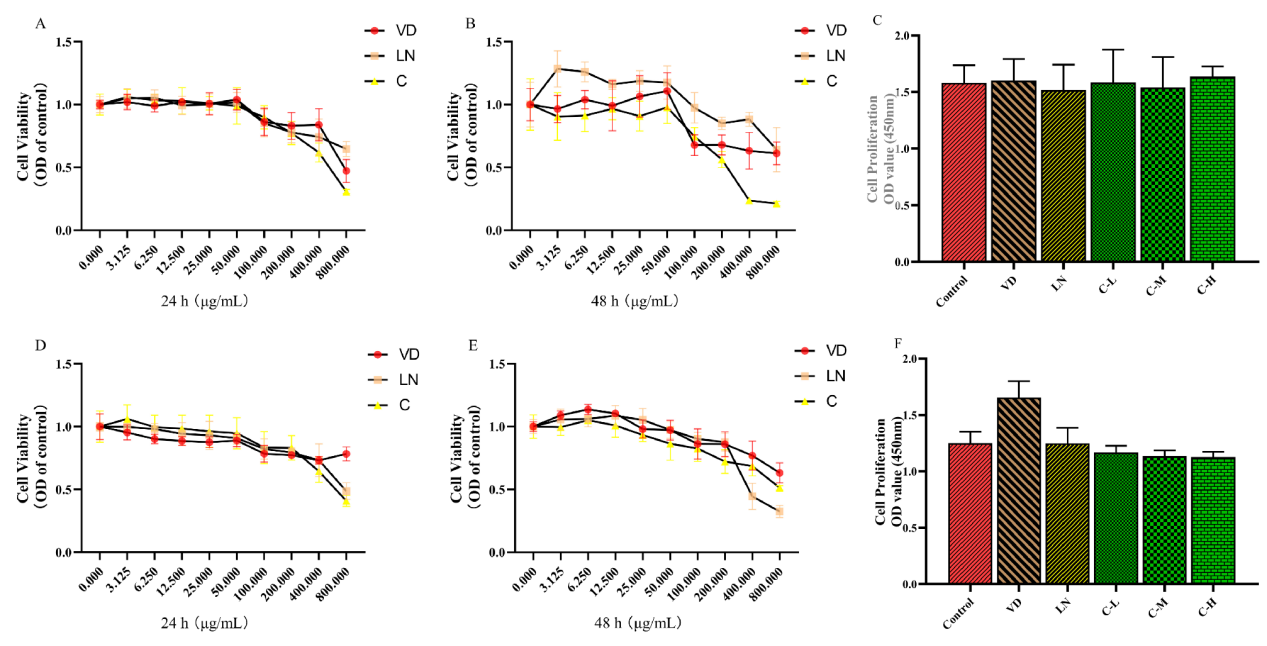
**

**Supplementary** **Fig. 1. Caffeine's effects on osteoclasts and osteoblasts.** CCK-8 analysis of Caffeine’s cytotoxicity in RAW264.7 cells at 24 h (A) and 48 h (B). Cell proliferation of RANKL-induced RAW264.7 cells after 5 days (C). CCK-8 analysis of Caffeine’s cytotoxicity in MC3T3-E1 cells at 24 h (D) and 48 h (E). Cell proliferation of L-ascorbic acid + β-glycerophosphate-induced MC3T3-E1 cells after 14 days (F). Experiments were repeated 3 times.

**
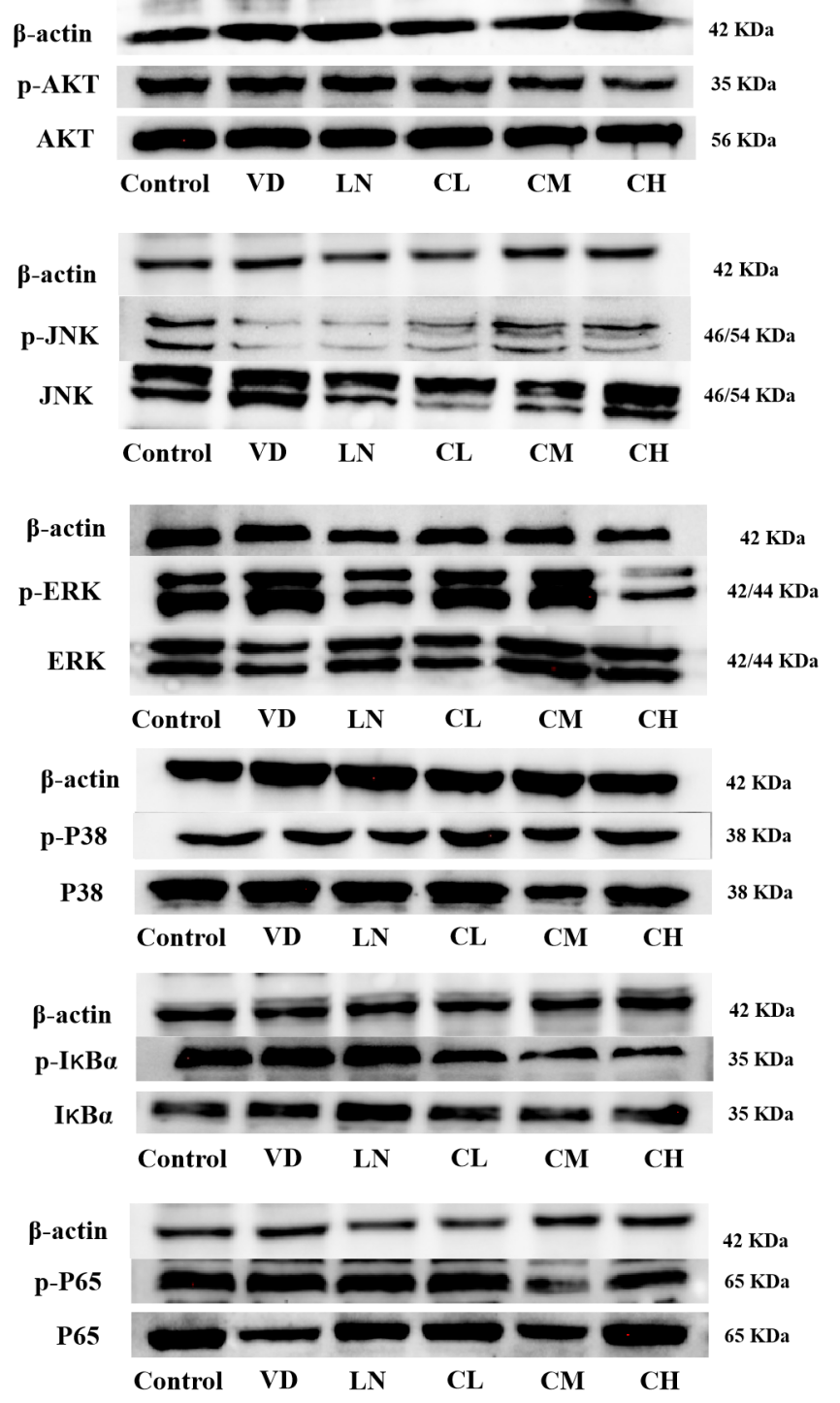
**

**Supplementary Fig. 2. Figure 2E-J internal controls.**

**
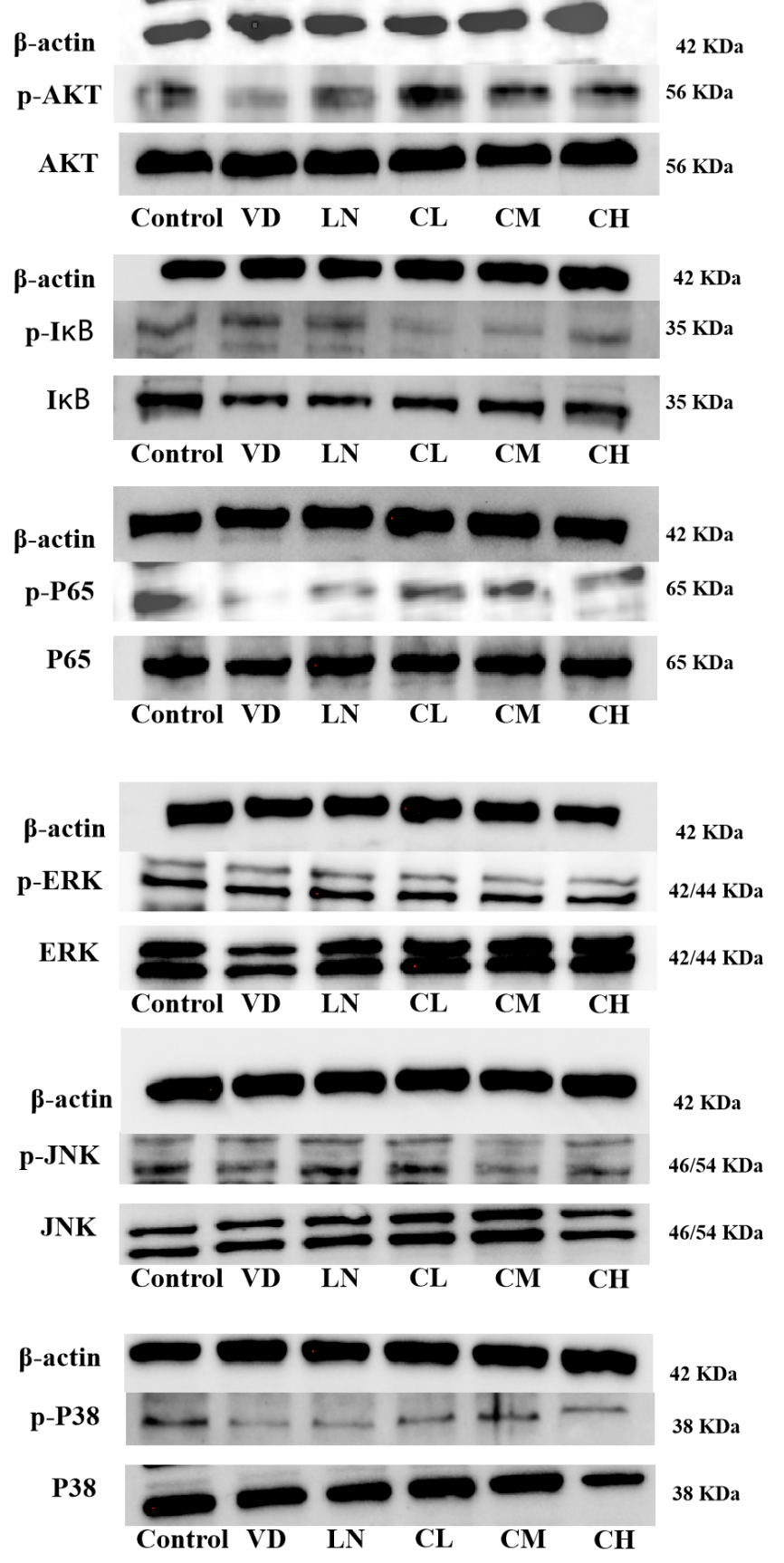
**

**Supplementary Fig. 3. Figure 3C-H internal controls.**
